# Depot-specific metabolic and inflammatory profiles in perirenal and renal sinus adipose tissue

**DOI:** 10.1186/s10020-025-01323-1

**Published:** 2025-07-22

**Authors:** Maria J. Pereira, Argyri Mathioudaki, Susanne Hetty, Amir Sedigh, Jan W. Eriksson, Maria K. Svensson

**Affiliations:** 1 Department of Medical Sciences, Clinical Diabetology and Metabolism, Uppsala University, Uppsala, Sweden; 2 Department of Surgical Sciences, Transplantation Surgery, Uppsala University, Uppsala, Sweden; 3 Present Address: Department of Medical Sciences, Renal Medicine, Uppsala University, Uppsala University Hospital, Uppsala, 751 85 Sweden; 4 Uppsala Clinical Research Center, Uppsala University, Uppsala, Sweden

**Keywords:** Omental adipose tissue, Kidney, Transcriptomics, Renal sinus adipose tissue, Perirenal adipose tissue, Adipocyte, Glucose uptake

## Abstract

**Background:**

Alterations in kidney-associated adipose tissue depots, specifically renal sinus (RSAT) and perirenal adipose tissue (PRAT), may contribute to metabolic, cardiovascular, and chronic kidney diseases. We compared transcriptomic profiles and phenotypes, including adipocyte size, glucose uptake, and insulin action in RSAT and PRAT from healthy individuals.

**Methods:**

Subcutaneous (SAT), omental (OAT) and renal adipose tissue biopsies were collected from healthy kidney donors (20 women, 20 men; BMI 20 to 36 kg/m^2^). Adipocyte size and basal and insulin-stimulated glucose uptake rate were measured in isolated adipocytes. Transcriptomic profiling and immune cell composition estimates (RNA seq, *n* = 30), were performed to evaluate differences between PRAT and RSAT, with OAT as a benchmark.

**Results:**

PRAT exhibited significantly larger adipocytes and higher insulin-stimulated glucose uptake than RSAT. Of 1113 significantly differentially expressed genes (DEGs) (PRAT: 571 down- and 542 upregulated), thermogenic and metabolic genes (*UCP1*, *CIDEA*, and *CKMT1B*) were enriched in PRAT, while inflammation-related genes (*NFKBIA*, *BIRC3*, and *IRF1*) in RSAT. Pathway analysis indicated activation of metabolic pathways (TCA cycle and oxidative phosphorylation), in PRAT, which contrasts with the immune and inflammatory pathways in RSAT and OAT. Immune cell gene signatures revealed an anti-inflammatory environment in PRAT (eosinophils and activated NK cells), and a pro-inflammatory profile in RSAT (M0 macrophages). Immunohistochemistry confirmed higher CD68- and IL1B-positive cells in RSAT than in PRAT. When overweight individuals were compared to lean, genes related to the VEGF signaling were upregulated in PRAT and Ras signaling in RSAT. Additionally, metabolic pathways linked to the TCA cycle as well as carbon and fatty acid metabolism were downregulated.

**Conclusions:**

The different kidney-associated adipose tissue depots exhibit distinct gene expression and functional profiles. PRAT displays higher expression of thermogenic markers and less inflammatory profile compared to RSAT and also OAT. In contrast, RSAT exhibits an inflammatory and macrophage-enriched profile, more closely resembling OAT. This study highlights the heterogeneity of the kidney-associated adipose tissue depots and could suggest that an excessive amount of RSAT may impact development of metabolic, cardiovascular, and chronic kidney diseases.

**Supplementary Information:**

The online version contains supplementary material available at 10.1186/s10020-025-01323-1.

## Background

Obesity is a major global health concern and a key risk factor for metabolic, cardiovascular and chronic kidney diseases. As the prevalence of obesity continues to rise, understanding the role of different adipose tissue depots in disease development becomes crucial. Previous studies have indicated that the anatomical location, quantity, and function of adipose tissue are significant factors.

Previously, the adipose tissue around the kidneys—renal sinus adipose tissue (RSAT) and perirenal adipose tissue (PRAT)—has been regarded primarily as mechanical support for the kidneys. However, recent studies suggest that these kidney-associated adipose tissue depots may actively contribute to the onset and progression of obesity-related metabolic, cardiovascular, and chronic kidney diseases [1–4].

Renal sinus adipose tissue (RSAT) has been linked to mechanical compression of renal structures, inflammatory processes, and detrimental effects on kidney function [5–8]. In contrast, perirenal adipose tissue (PRAT) is composed of brown, beige and white adipocytes, with brown adipocytes playing a crucial role in thermogenesis and energy expenditure [4]. Perirenal fat surrounds the kidneys and adrenal glands, whereas RSAT represents an anatomical extension of PRAT originating around the renal hilum. There is no distinct boundary separation between these depots, and RSAT is often considered a part of perirenal fat [9]. Despite their anatomical proximity, these adipose depots have been shown to have distinct characteristics. However, their specific roles in obesity-related diseases remain incompletely understood.

Previous research has focused on characterizing these adipose tissue depots individually, emphasizing the metabolic activity of PRAT, as a brown adipose tissue (BAT)-enriched depot [10]. Our research group recently highlighted the inflammatory nature of RSAT [5], demonstrating that RSAT more closely resembles omental adipose tissue (OAT) than SAT at both the molecular and functional levels. However, no direct comparison between RSAT and PRAT has previously been conducted. This study therefore aimed to compare the phenotypic and transcriptomic profiles of RSAT and PRAT from healthy individuals. By analyzing adipocyte size, glucose uptake rates, and gene expression profiles, including those of brown and beige adipose tissue-specific markers, we evaluated functional differences between these two adipose tissue depots.

## Methods

### Subjects and sample collection

Adipose tissue samples from two distinct renal depots, RSAT and PRAT, were obtained from 40 healthy individuals with normal blood pressure, kidney function, and fasting metabolic parameters during laparoscopic unilateral nephrectomy for kidney donation at Uppsala University Hospital, as previously reported [5]. All adipose tissue samples were carefully rinsed with saline solution to minimize residual blood. For comparative analyses, omental (OAT) and subcutaneous adipose tissue (SAT), previously well characterized fat depots, were also collected.

Some of the adipose tissue samples were snap-frozen in liquid nitrogen and stored at −80 °C for subsequent mRNA isolation and transcriptomic analyses (*n* = 30). Additionally, mature adipocytes were isolated for cell size measurements and ex vivo glucose uptake rate analyses. Due to limited tissue amounts, not all analyses could be performed on all samples. Fasting blood samples were collected for analyses of HbA1c, plasma glucose, serum insulin, and C-peptide. Kidney function was preoperatively assessed using both the creatinine-based estimated glomerular filtration rate (eGFR) [11] and iohexol clearance (mGFR) [12]. The anthropometric and clinical characteristics of the study participants, as previously described [5], included 20 women and 20 men with a mean age 50 ± 11 years, BMI 26.4 ± 3.5 kg/m^2^, body fat mass% 29.3 ± 9.5%, mGFR 97.8 ± 11.8 ml/min and eGFR 82.3 ± 8.6 ml/min/1.73m^2^ (additional File 2 Table S1). All study participants provided written consent, and the Swedish Ethical Review Authority approved the study with dnr 2020–06350. All study procedures were performed following the Declaration of Helsinki.

### Adipocyte glucose uptake rate and cell size

Mature adipocytes were used for adipocyte size measurements (PRAT *n* = 30, RSAT *n* = 33, OAT *n* = 31, and SAT *n* = 38) and ex vivo glucose uptake rate assessments (PRAT *n* = 26, RSAT *n* = 13, OAT *n* = 15, and SAT *n* = 18). Adipocyte size was determined by measuring the diameters of 100 individual cells using brightfield microscopy. Glucose uptake was quantified under both basal and insulin-stimulated conditions (25 and 1000 µU/mL) following incubation with [^14^C]-glucose using previously published methods [13, 14].

### RNA extraction, sequencing, and analyses

Total RNA was extracted from the adipose tissue depots using the TRIzol protocol [15, 16]. High quality RNA samples (RIN > 5) from RSAT (*n* = 28), PRAT (*n* = 28), and OAT (*n* = 27) were processed for library preparation and sequencing by Novogene Co., Ltd. (Cambridge, UK). The raw reads in *fastq* format were preprocessed with *fastp* (version 1.0.8) to remove adapters and low-quality reads. The cleaned paired-end reads were mapped to the GRCh38 reference genome using Hisat2 (version 2.0.5). Quality control checks following alignment were conducted with MultiQC [17]. The raw counts were normalized to counts per million (CPM) using *edgeR* (version 3.42.4) [18]. Genes with low expression were filtered out (additional File 1 Figure S1**)**, and normalization was performed using the trimmed mean of M-values method in edgeR. Sample similarities were assessed using multi-dimensional scaling plots generated with limma (version 3.56.2) [19], and outliers were removed (additional File 1 Figure S2).

### Differential expression analysis and functional enrichment analysis

Differential gene expression analyses were conducted between adipose tissue depots overall (PRAT vs. RSAT and PRAT vs. OAT), as well as stratified by BMI category: overweight (BMI ≥ 25 kg/m^2^, *n* = 18) and normal-weight individuals (BMI < 25 kg/m^2^, *n* = 12). Comparisons between RSAT vs. SAT and OAT vs. SAT were previously conducted and reported in our previous publication [5]. A linear modelling approach was used with the *limma* and *voom* [19, 20] R packages (additional File 1 Figure S3**)**. P-values were adjusted using the Benjamini & Hochberg method with significance set at p-value < 0.05. Differentially expressed genes (DEGs) underwent functional enrichment analysis using the R package g: Profiler [21] to identify enriched biological pathways and processes. Additionally, the expression levels of BAT-specific beige and white adipose tissue markers were analyzed, with marker genes selected on the basis of previous reports in the literature that has identified these genes as specific markers of distinct adipocyte phenotypes [22, 23] across the PRAT, RSAT, and OAT depots. Markers of neural growth, macrophages and inflammatory markers were also investigated in these depots.

### Weighted gene co-expression network analysis (WGCNA)

To identify modules of co-regulated genes, the weighted gene co-expression network analysis (WGCNA) framework was applied to the PRAT depot. RNA-seq data from PRAT samples were used after low-expressed genes (requiring at least 15 counts in at least 75% of samples) were filtered and the data normalized. A matrix of expression values suitable for network construction was obtained. An appropriate soft-thresholding power of *12* to ensure a scale-free topology was then selected, constructing a signed weighted network using the *blockwiseModules* function within the WGCNA package in R [24], which clusters genes into modules on the basis of their topological overlap. Using Pearson correlation coefficients, a weighted adjacency matrix was created, leading to distinct gene modules (additional File 1 Figure S4). Once the modules were identified, their eigengenes (the first principal component of each module) were computed and these module eigengenes were correlated with relevant clinical and biochemical characteristics (e.g., body composition (BMI, WHR, fat mass), glucose, HOMA-IR, blood pressure, and kidney function (mGFR)). Modules showing the strongest positive or negative correlations with clinical parameters were further investigated with pathway enrichment analysis.

### Analysis of cell type deconvolution

To estimate the immune cell composition of the adipose tissue depots, cell-type deconvolution of the RNA-seq data using CIBERSORTx algorithm was performed [25]. The LM22 signature matrix was used as reference for the deconvolution analysis (additional File 2 Table S2). This includes 547 genes and can efficiently distinguish 22 mature human hematopoietic cell populations—including T-cell subsets (e.g., CD4 memory and regulatory T cells), B cells, plasma cells, natural killer (NK) cells, and myeloid subsets (e.g., macrophages, monocytes, and neutrophils). This combined reference and algorithm with default parameters for RNA seq data were subsequently used to generate relative abundance scores and average fractions for each immune cell type for each adipose tissue depot. To evaluate overall immune activity, the fractions were aggregated by tissue type, and the average fraction of each immune cell type was calculated for each depot. Dominant immune cell types were identified, and significant variations in immune cell composition across PRAT, RSAT, and OAT were assessed using Wilcoxon rank-sum test and FDR to adjust for multiple statistical comparisons.

### Immunohistochemistry

Formalin-fixed, paraffin-embedded paired PRAT, RSAT and OAT samples from two subjects were sectioned (5 μm) and placed on Superfrost Plus Adhesion Microscope Slides (Epredia). Adipose tissue sections were deparaffinized in xylen, rehydrated in ethanol (decreasing concentrations from 100 − 70%) and rinsed in distilled water. Endogenous hydrogen peroxide activity was quenched in 0.3% hydrogen peroxide for 15 min, followed by heat-induced antigen retrieval in 10 mM sodium citrate buffer (pH 6.0) for 30 min in a water bath brought to boiling point. The sections were blocked for 20 min in either 10% normal goat serum or normal horse serum, depending on the secondary antibody used, in PBS with 0.05% Triton X-100. Primary antibody incubation was performed overnight at 4 °C in a humid chamber with the anti-CD68 (dilution 1:100, Cat no. 916104, Biolegend, RRID: AB_2616797), anti-IL1b (dilution 1:100, Cat no. P420B, Invitrogen, RRID: AB_223478), and anti-UCP1 (concentration 10 µg/mL, Cat# MAB6158, Antibody R&D Systems, RRID: AB_10572490) antibodies. Control tissue sections were similarly prepared, but the primary antibody was omitted. The slides were then incubated with secondary biotinylated anti-rabbit or biotinylated anti-mouse antibodies (Cat no. BA-1000, RRID: AB_2313606 and Cat no. BA-2000, RRID: AB_2313581, respectively, Vector Laboratories) for 30 min at room temperature in a humidified chamber. Staining was performed via a 30 min incubation with VECTASTAIN ABC Kit, followed by incubation with the DAB Peroxidase Substrate kit for 8 min (both Vector Laboratories, California, USA) according to the manufacturers’ manuals. The sections were then dehydrated by washing with ethanol (increasing concentrations from 70 to 100%) followed by xylene, and sealed with PERTEX mounting medium (Histolab). Imaging was performed with a 20x objective on a Zeiss Axio Imager Z1 (NY, USA).

### Statistical analysis

In this exploratory hypothesis-generating study, data is presented as means ± SEM unless otherwise stated. Normality was assessed using the Shapiro-Wilk test and histograms. All correlations were calculated using Spearman correlation. Differences in adipocyte size and glucose uptake were assessed in paired samples analyses with linear mixed models, with individuals included as random effects to account for intra-individual correlations. Multiple comparisons between groups were corrected using the FDR adjustment method of Benjamin, Krieger, and Yekutieli. Gene expression values were log-transformed to stabilize variance and values of zero were substituted with 0.01 prior to transformation. Comparisons of mean gene expression across depots were performed using the Wilcoxon rank-sum test with FDR adjustment for multiple comparisons. Data visualization and statistical analyses were conducted in R version 4.3.2 using packages *ggplot2* (version 3.4.3) and *tidyplots* [26, 27], as well as GraphPad Prism version 10.4.2. Significance levels are denoted as *p < 0.05; **p < 0.01; ***p < 0.001.

## Results

### Comparison of adipocyte size across adipose depots

**Fig. 1:**
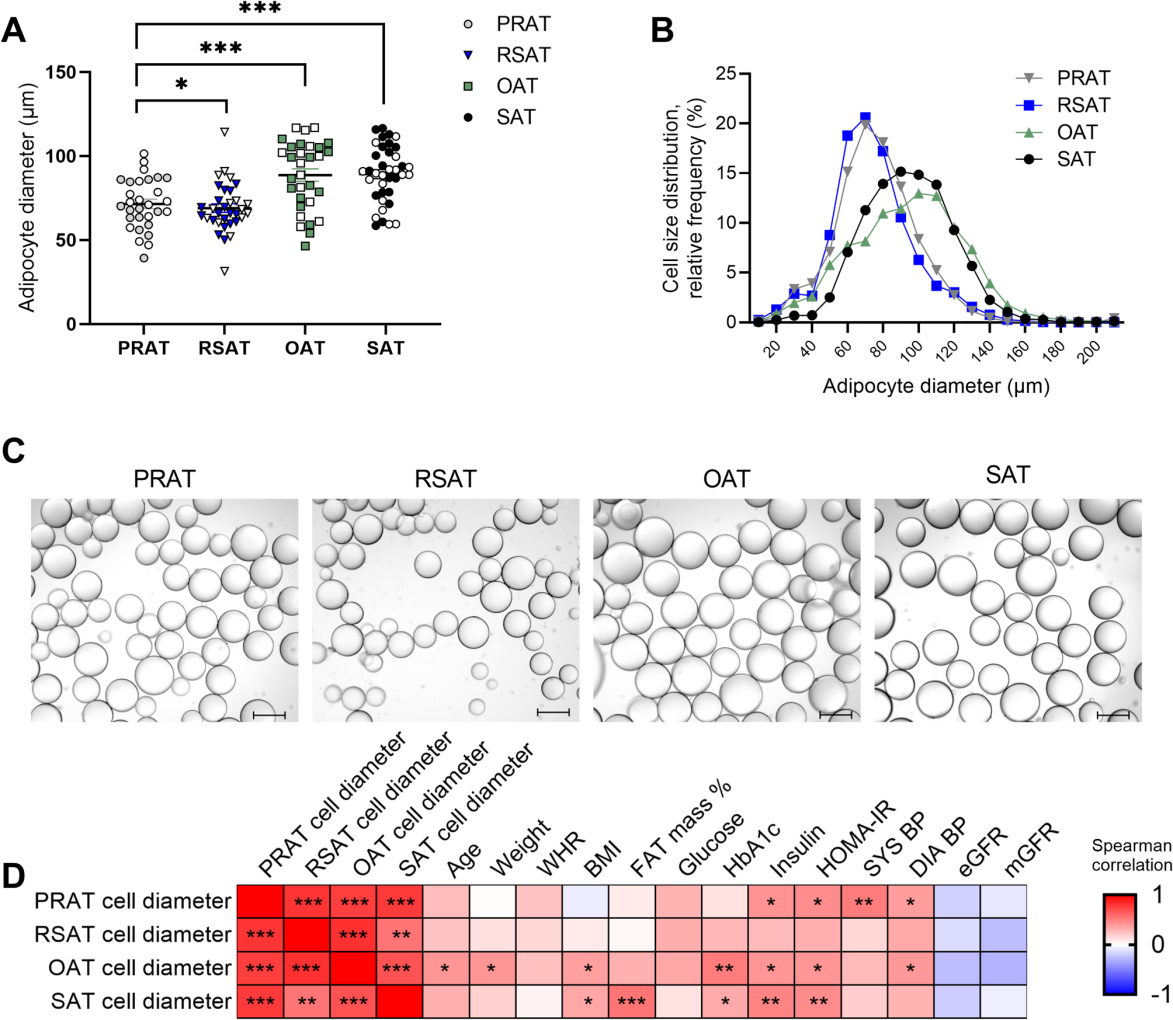
Adipocyte size distribution across four adipose tissue depots; perirenal adipose tissue (PRAT), renal sinus adipose tissue (RSAT), omental adipose tissue (OAT) and subcutaneous adipose tissue (SAT). **A** Quantitative representation of average adipocyte size (PRAT: 71.5 ± 2.7 μm, RSAT: 69.0 ± 2.5 μm, OAT: 88.7 ± 3.7 μm, and SAT: 89.3 ± 2.7 μm). Open symbols represent males; filled symbols represent females. **B** Cell size distribution calculated as the relative frequency (%) for each adipose tissue depot; (**C**) Representative image obtained by light microscopy at 10x magnification. **D** Heatmap showing correlation between adipocyte size in PRAT and clinical and biochemical characteristics of study participants. Correlations range from − 1 (red) to + 1 (blue). WHR; Waist-to-Hip Ratio, BMI; Body mass index (kg/m2), HbA1c; Hemoglobin A1c, HOMA-IR; Homeostatic Model assessment of Insulin resistance, SYS BP; Systolic blood pressure, DIA BP; Diastolic blood pressure, eGFR; estimated glomerular filtration rate (creatinine-based), mGFR; measured glomerular filtration rate (iohexol clearance). Bar = 100 μm, PRAT: *n* = 30, RSAT: *n* = 33, OAT: *n* = 31, SAT: *n* = 38. *p < 0.05; **p < 0.01; ***p < 0.001

This manuscript focuses on the characterization of PRAT, whereas comparisons of adipocyte size in RSAT, OAT, and SAT, along with their clinical correlations, have been reported previously [5]. PRAT adipocyte size was smaller compared to OAT (*p* < 0.001) and SAT (*p* < 0.001), but larger than RSAT (*p* < 0.05) (Fig. 1A-C**).** PRAT, OAT and SAT adipocyte size was positively correlated with metabolic parameters (insulin levels, HOMA-IR) and blood pressure (systolic and diastolic) (Fig. 1D and additional File 2 Table S3). PRAT and RSAT did not correlate with markers of body composition (Fig. 1D) or differ between overweight and lean subjects (additional File 1 Figure S5). Furthermore, PRAT adipocyte size correlated significantly with RSAT, OAT and SAT adipocyte size. Consistent with our previous findings there was no sex differences in SAT, OAT, and RSAT [5] and PRAT adipocyte size.

To evaluate whether PRAT adipocyte size is an independent predictor of HOMA-IR and fasting plasma insulin, even when adjusted for RSAT or OAT adipocyte size, we performed multiple linear regression analyses (RSAT and OAT in independent models to avoid multicollinearity) (additional File 2 Table S4). In models adjusting for RSAT adipocyte size, PRAT adipocyte size remained significantly associated with HOMA-IR (stand β = 0.625, *p* = 0.034) and fasting insulin (stand β = 0.626, *p* = 0.035). In models adjusting for OAT adipocyte size, PRAT adipocyte size showed a positive but non-significant trend for HOMA-IR (stand β = 0.453, *p* = 0.102) and insulin (stand β = 0.453, *p* = 0.102).

### Comparison of glucose uptake rates across adipose tissue depots

**Fig. 2:**
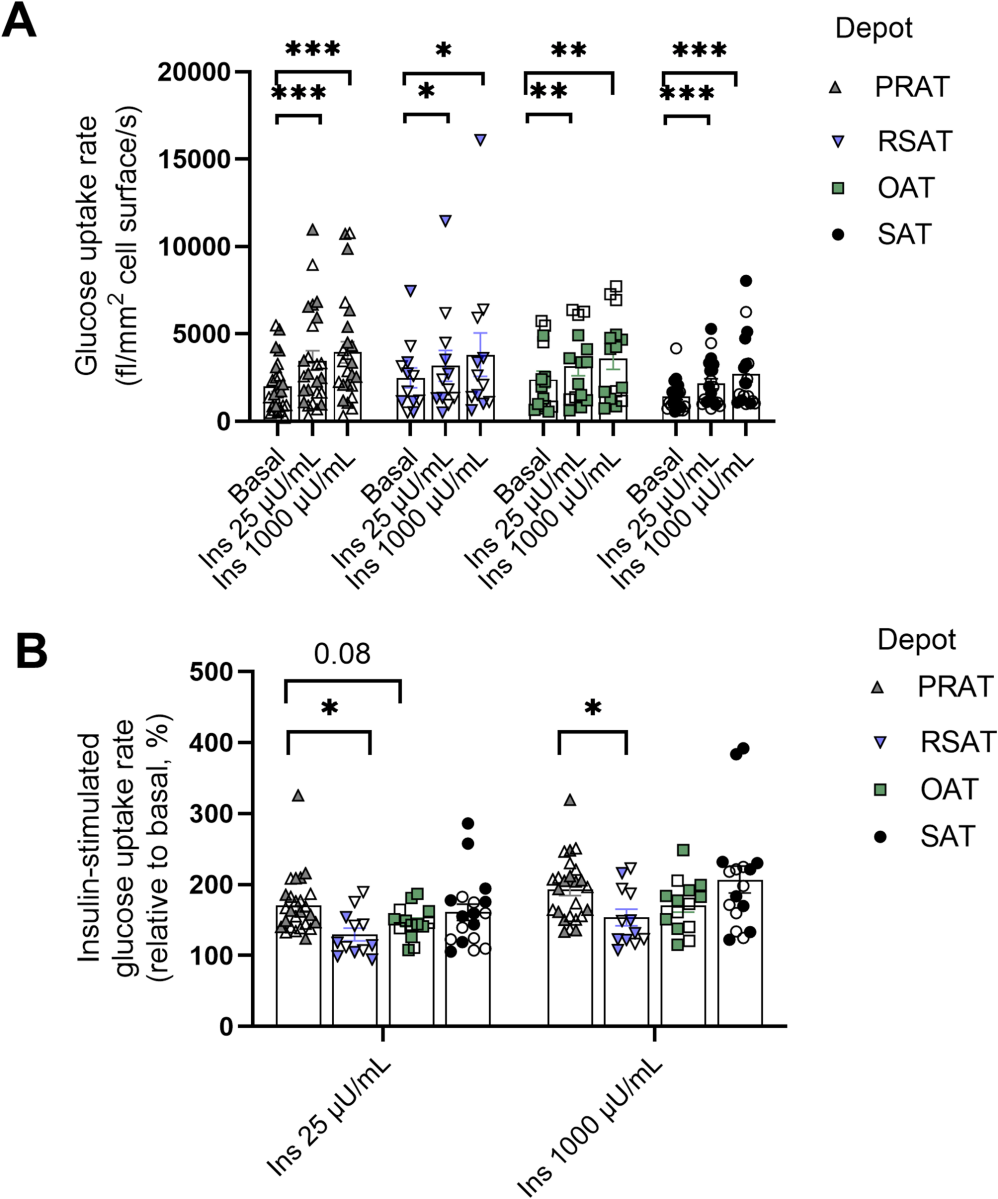
Adipocyte glucose uptake profile across four adipose tissue depots; perirenal adipose tissue (PRAT), renal sinus adipose tissue (RSAT), omental adipose tissue (OAT) and subcutaneous adipose tissue (SAT). **A** Adipocyte glucose uptake rate normalized to the total cell surface area, under basal conditions and after insulin stimulation (25 and 1000 µU/mL) in the PRAT (gray), RSAT (blue), OAT (green), and SAT (black) groups. **B** Incremental glucose uptake response rate to insulin stimulation (25 and 1000 µU/mL) relative to basal levels, with that of the PRAT. PRAT: *n* = 25, RSAT: *n* = 12, OAT: *n* = 14, SAT: *n* = 18. *p < 0.05; **p < 0.01, ***p < 0.001

Compared with basal conditions, insulin significantly stimulated glucose uptake in all adipose tissue depots; PRAT, RSAT, OAT, and SAT (*p* < 0.05 for all) (Fig. 2A). No significant differences in glucose uptake levels were found across the depots under either basal or insulin-stimulated conditions. PRAT demonstrated a greater glucose uptake response to insulin at both 25 and 1000 µU/mL compared to RSAT (*p* < 0.05, Fig. 2B) and showed a tendency to enhance insulin responsiveness at 25 µU/mL insulin compared to OAT (*p* = 0.08). PRAT glucose uptake did not differ between subjects with and without overweight, but in RSAT, both basal and insulin-stimulated glucose uptake (at 25 and 1000 µU/mL insulin) showed a trend toward lower values in overweight individuals compared to lean (*p* = 0.06) (additional File 1, Figure S5).

### Comparison of brown, beige, and white adipose tissue-specific markers in different adipose tissue depots

**Fig. 3:**
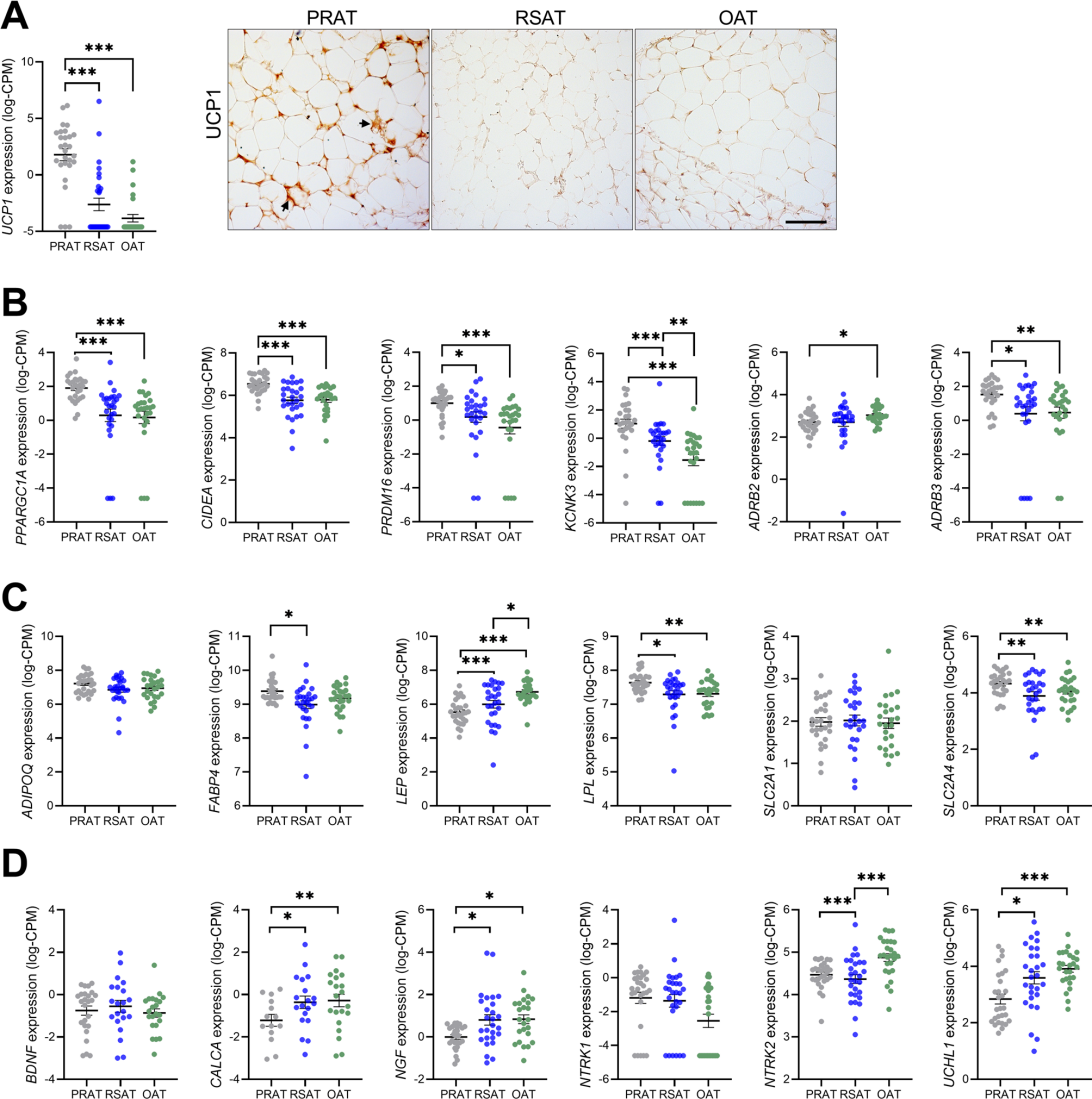
Expression of brown/beige, white adipose tissue-specific markers and neural markers across three adipose tissue depots: perirenal adipose tissue (PRAT), renal sinus adipose tissue (RSAT), and omental adipose tissue (OAT). The expression levels are presented as log counts per million (logCPM). **A** Gene expression and immunohistochemistry of *UCP1* (uncoupling protein 1). Positive staining (brown) indicates UCP1 staining. Images were acquired at 20× magnification; scale bar = 100 μm. **B**) Overlap of brown and beige adipose tissue markers (BeAT and BAT): The panel shows expression of *CIDEA* (cell death-inducing DFFA-like effector a); *KCNK3* (potassium channel subfamily K member 3); PRDM*16* (PR domain containing 16); *PPARGC1A* (peroxisome proliferator-activated receptor gamma coactivator 1-alpha); *ADRB2* (beta-2 adrenergic receptor); *ADRB3* (beta-3 adrenergic receptor). **C** The panel shows expression of white adipose tissue (WAT) markers and functionally related genes: *ADIPOQ* (adiponectin); *FABP4* (fatty acid-binding protein 4); *LPL* (lipoprotein lipase); *LEP* (leptin); *SLC2A1* (GLUT1); and *SLC2A4* (GLUT4). **D** The panel shows expression of *BDNF* (brain-derived neurotrophic factor); *CALCA* (calcitonin-related polypeptide alpha); *NGF* (nerve growth factor); *NTRK1* (neurotrophic receptor tyrosine kinase 2); *NTRK2* (neurotrophic receptor tyrosine kinase 2); *UCHL1* (ubiquitin carboxy-terminal hydrolase L1). PRAT: *n* = 28, RSAT: *n* = 28, OAT: *n* = 26. Significance levels after FDR adjustment *p < 0.05, **p < 0.01, ***p < 0.001

The expression of markers specific to brown, beige, and white adipocytes was analyzed to assess depot-specific characteristics (PRAT, RSAT, and OAT) (Fig. 3). Both gene expression and immunohistochemistry revealed significantly higher UCP1 (uncoupling protein 1) levels in PRAT compared to RSAT and OAT (*p* < 0.001) (Fig. 3A). PRAT consistently presented higher *UCP1* gene expression levels, with moderate variation between individuals (additional File 1 Figure S6). RSAT had low or no *UCP1* expression, with peaks only in a few individuals (*n* = 4). In OAT, *UCP1* expression levels remain low, with minimal variability. PRAT also showed increased expression of additional brown/beige adipocyte markers, including *CIDEA* (cell death-inducing DNA fragmentation factor alpha-like effector A), *PRDM16* (PR domain-containing 16), *KCNK3* (potassium channel subfamily K member 3), *ADRB3* (beta-3 adrenergic receptor), and *PPARGC1A* (peroxisome proliferator-activated receptor gamma coactivator 1-alpha) (*p* < 0.05) (Fig. 3B). Only *ADRB2* (beta-2 adrenergic receptor) was expressed at significantly higher levels in OAT compared to PRAT (*p* < 0.05). In contrast, white adipose tissue (WAT)-specific markers (Fig. 3C) displayed different expression patterns across adipose tissue depots. No significant difference was observed for *ADIPOQ* (adiponectin), whereas *LEP* (leptin) was more highly expressed in OAT, than in PRAT and RSAT (*p* < 0.01). *FABP4* (fatty acid-binding protein 4) was expressed at higher levels in PRAT than in RSAT (*p* < 0.05) and the expression of *LPL* (lipoprotein lipase) was significantly higher in PRAT than in both RSAT (*p* < 0.05) and OAT (*p* < 0.01). Additionally, *SLC2A4* (*GLUT4*) gene expression, but not *SLC2A1* (*GLUT1*), was higher in PRAT compared to OAT and RSAT (Fig. 3C). To further explore the neuronal regulation of adipose tissue, the expression of neurotrophic and neuronal markers was assessed (Fig. 3D). Compared to PRAT and RSAT, OAT showed higher expression of *CALCA* (calcitonin-related polypeptide alpha), *NGF* (nerve growth factor), *NTRK2* (neurotrophic receptor tyrosine kinase 2), and *UCHL1* (ubiquitin carboxy-terminal hydrolase L1) (*p* < 0.05). No significant differences were observed for *BDNF* (brain-derived neurotrophic factor) or *NTRK2* (neurotrophic receptor tyrosine kinase 2) between the adipose tissue depots. To assess potential differences in tissue vascularization, we examined expression of several vascular markers *PECAM1* (platelet/endothelial cell adhesion molecule 1, also known as CD31), *CDH5* (cadherin 5, also known as VE-cadherin or CD144), *VEGFA* (vascular endothelial growth factor A), and *VWF* (von Willebrand factor). No significant depot-specific differences were observed in the expression of these markers (additional File 1 Figure S7).

### Comparative transcriptomic analysis across adipose tissue depots

**Fig. 4:**
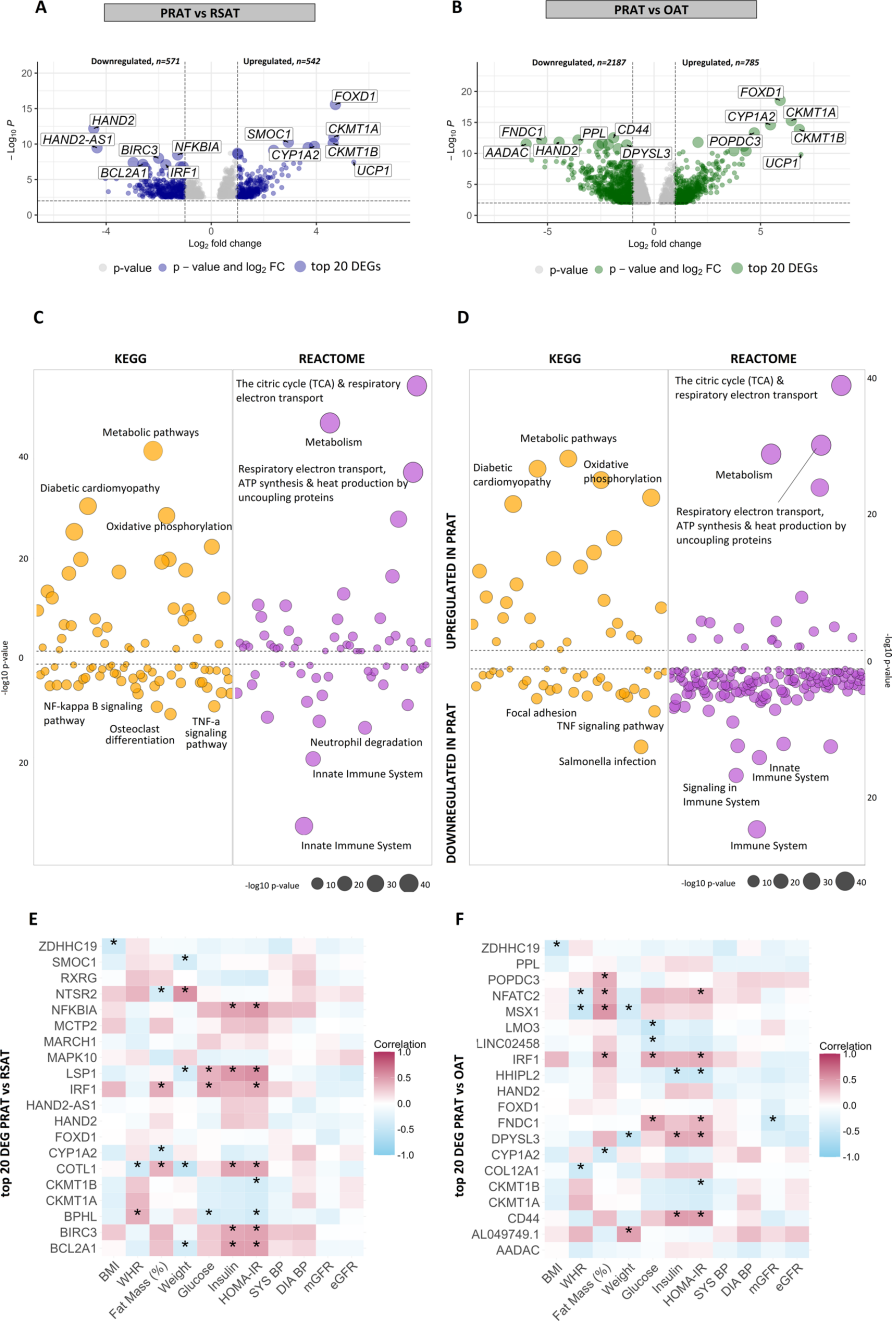
Differential gene expression and pathway enrichment analysis in perirenal adipose tissue (PRAT) compared with renal sinus adipose tissue (RSAT) and omental adipose tissue (OAT). Volcano plot showing differentially expressed genes (DEGs) that are downregulated (left) or upregulated (right) in (**A**) PRAT compared with RSAT and (**B**) PRAT compared with OAT. The x-axis represents the log2 fold change, whereas the y-axis shows the -log10 p-value. The genes with larger dots represent the top 20 DEGs with *p*-values, 10 upregulated and 10 downregulated. **C** Pathway enrichment analysis of upregulated (top) and downregulated (bottom) KEGG and REACTOME pathways in PRAT compared with RSAT (**C**) and PRAT compared with OAT (**D**). The x-axis represents the pathway categories, whereas the y-axis shows the -log10 (adjusted *p*-value) for each pathway. Correlations were performed between the top 20 DEGs identified between PRAT and RSAT (**E**) and between PRAT and OAT (**F**) and clinical characteristics. BMI; Body mass index (kg/m2), WHR; Waist to Hip ratio, HOMA-IR; Homeostatic Model assessment of Insulin resistance, SYS BP; Systolic blood pressure, DIA BP; Diastolic blood pressure, mGFR; measured glomerular filtration rate (iohexol clearance); and eGFR; estimated glomerular filtration rate. PRAT: *n* = 28, RSAT: *n* = 28, OAT: *n* = 26. *p < 0.05

The transcriptomic profiles of PRAT, RSAT, and OAT were investigated via differential expression and pathway enrichment analyses (Fig. 4). A total of 1,113 significantly differentially expressed genes (DEGs) were identified between PRAT and RSAT, with 571 genes downregulated and 542 genes upregulated in PRAT (Fig. 4A and additional File 2 Table S5). Similarly, when PRAT was compared with OAT, 2,972 DEGs were identified, of which 2,187 were downregulated and 785 were upregulated in PRAT (Fig. 4B and additional File 2 Table S5). The considerably smaller number of DEGs in the comparison between PRAT and RSAT shows that these two depots share more similar gene expression profiles, i.e., are more similar, then when PRAT is compared with OAT.

The levels of thermogenic and metabolic markers, including *UCP1*, *FOXD1*, *CKMT1A/1B*, and *CYP1A2*, were consistently higher in PRAT than in RSAT and OAT. Conversely, genes involved in immune regulation and inflammation, such as *HAND2*, *BIRC3*, and *NFKBIA* were significantly lower in PRAT than in RSAT, and structural and functional remodeling, such as *PPL*,* FNDC1*,* CD44*, and *AADAC* were significantly lower in PRAT compared with OAT.

Pathway enrichment analysis was used to further elucidate the functional differences between PRAT and the other adipose tissue depots (additional File 2 Table S6). KEGG and Reactome annotation of the enriched pathways revealed an increase in metabolic pathways, TCA and respiratory electron transport, and oxidative phosphorylation in PRAT compared to RSAT and OAT, (Figs. 4C and D). In contrast, pathways involved in immune system activity, e.g., the NF-kappa B and TNF-alpha signaling pathways and neutrophil degradation, were downregulated in PRAT compared with RSAT (Fig. 4C), and TNF-alpha signaling, the immune system and the innate immune system were downregulated in PRAT compared with OAT (Fig. 4D).

### Correlations between clinical characteristics and gene expression across adipose tissue depots

The top ten upregulated and downregulated DEGs in PRAT, from both comparisons, i.e., with RSAT and OAT, were correlated with several clinical characteristics, including body composition (BMI, WHR, fat mass %), insulin resistance markers (glucose, insulin, HOMA-IR) and systolic blood pressure (Fig. 4E and F and additional File 2 Table S7). In the PRAT-to-RSAT comparison, several genes, such as *LSP1* (lymphocyte-specific protein 1), *IRF1* (interferon regulatory factor 1), *COTL1* (coactosin-like protein 1), *BIRC3* (baculoviral inhibitor of apoptosis repeat-containing protein 3), and *BCL2A1* (B-cell lymphoma 2 related protein A1), were positively correlated with insulin resistance markers (glucose, insulin, HOMA-IR), and body composition (fat mass %) (Fig. 4E). Similarly, in the PRAT-to-OAT comparison, genes such as *IRF1* (interferon regulatory factor 1), *FNDC1* (fibronectin type III domain-containing protein 1), *DPYSL3* (dihydropyrimidinase-like protein 3), and *CD44* (cluster of differentiation 44) were positively correlated with markers of insulin resistance (glucose, insulin, and HOMA-IR) (Fig. 4F). Notably, many of the correlations with fat mass % were no longer significant after adjusting for HOMA-IR. This suggests that these gene expression patterns are more closely related to insulin resistance than adiposity per se (data not shown). *FNDC1* was also negatively correlated with iohexol clearance (mGFR), but no significant associations were found with blood pressure (systolic or diastolic) or estimated kidney function (eGFR).

### Comparative transcriptomics of PRAT and RSAT in overweight compared with normal-weight individuals

**Fig. 5:**
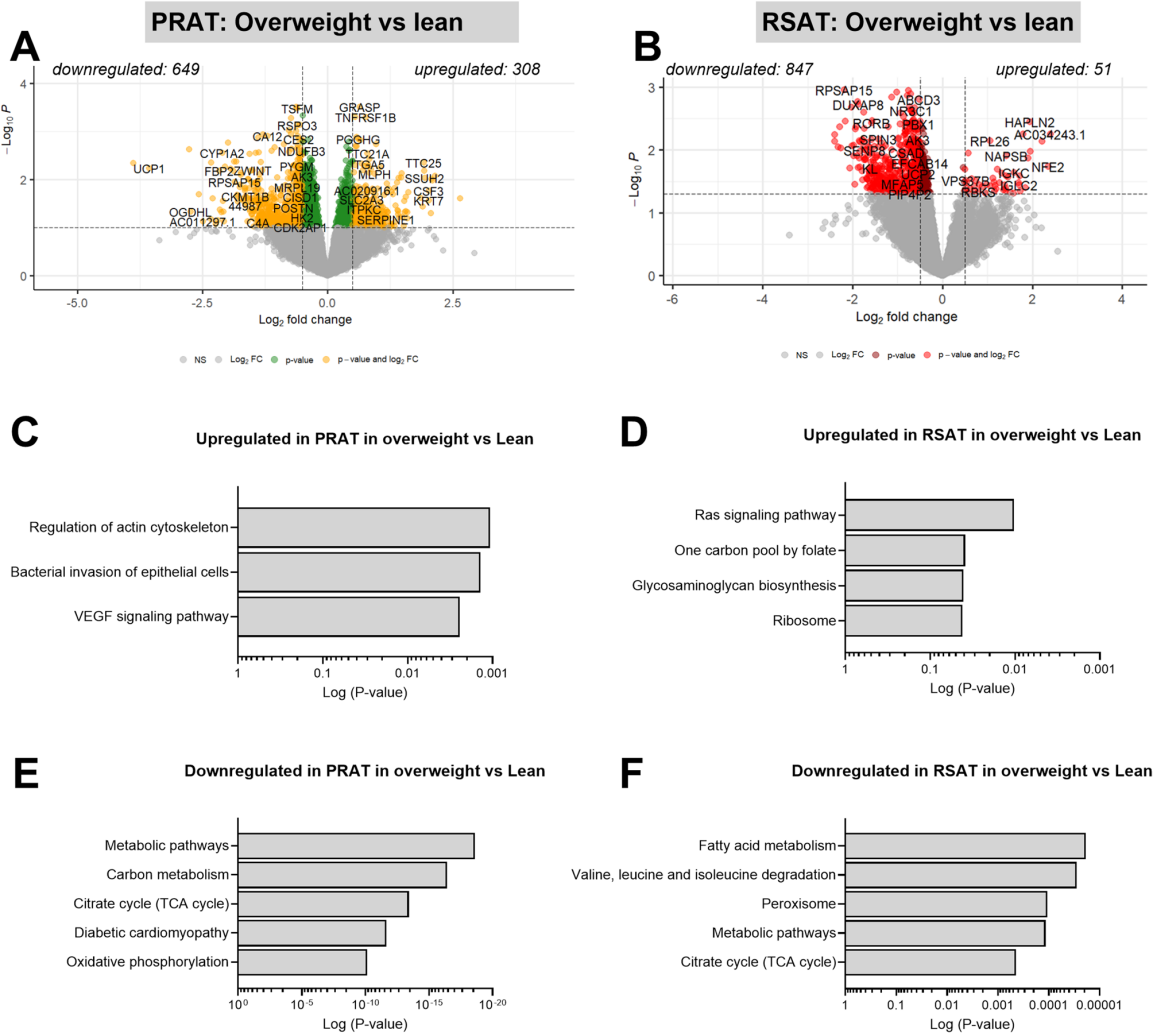
Differential gene expression and KEGG pathway enrichment analysis comparing overweight vs. normal-weight subjects in perirenal adipose tissue (PRAT) and renal sinus adipose tissue (RSAT). **A**, **B** Volcano plots showing differentially expressed genes (DEGs) in (A) PRAT and (B) RSAT. Each point represents a gene, with the x-axis indicating the log2 fold change and the y-axis showing the–log10 (unadjusted p-value). Genes to the left are downregulated, whereas those to the right are upregulated in overweight relative to normal-weight subjects. KEGG pathway enrichment of significantly upregulated (**C**, **D**) and downregulated (**E**, **F**) genes in PRAT and RSAT, respectively. (Normal-weight, PRAT: *n* = 10, RSAT: *n* = 11, OAT: *n* = 12; Overweight, PRAT: *n* = 18; RSAT: *n* = 17; OAT: *n* = 14). Pathways are ranked by statistical significance, with bars indicating the log10(p-value)

To explore the impact of excess weight on gene expression in PRAT and RSAT, we investigated the DEGs in these depots between overweight and normal-weight donors. In PRAT, 308 genes were upregulated and 649 were downregulated in overweight compared with normal-weight donors (Fig. 5A and additional File 2 Table S8). In RSAT, 51 genes were upregulated, and 847 were downregulated in overweight compared with normal-weight donors (Fig. 5B and additional File 2 Table S9). However, and importantly, these differences did not remain significant after FDR correction. For explorative purposes, we performed KEGG pathway analyses of the identified genes. In PRAT, genes related to the regulation of the actin cytoskeleton and the VEGF signaling pathway were upregulated in overweight individuals, whereas genes involved in carbohydrate metabolism, the TCA cycle, and oxidative phosphorylation were downregulated (Fig. 5C and E). In RSAT, genes related to the Ras signaling pathway and ribosome were upregulated in overweight individuals, whereas pathways related to fatty acid metabolism; valine, leucine, and isoleucine degradation; and the TCA cycle were downregulated (Fig. 5D and F).

### Weighted gene co-expression analysis (WGCNA) across adipose tissue depots

**Fig. 6:**
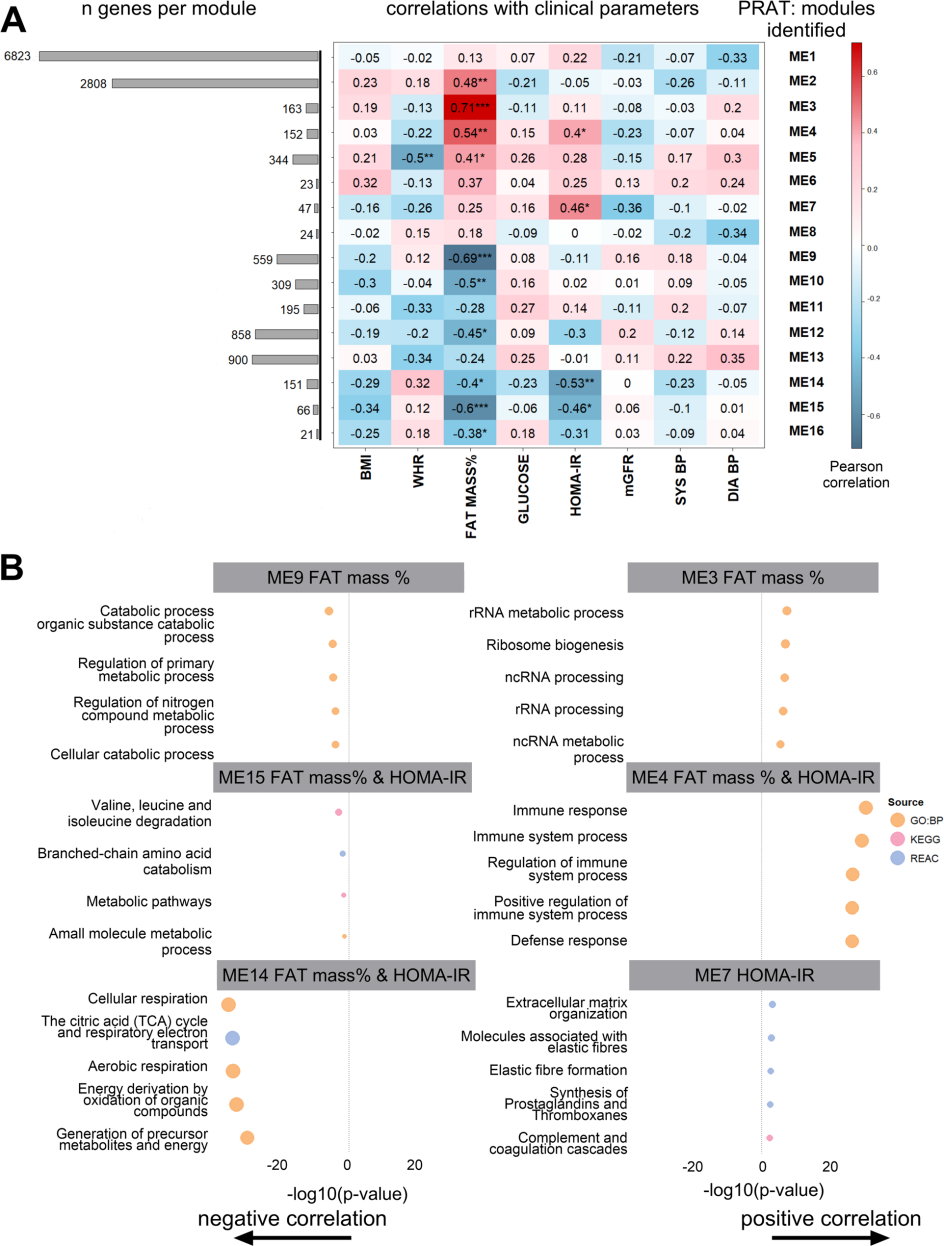
Correlation of gene networks from WGCNA in perirenal adipose tissue (PRAT) with clinical characteristics and pathway enrichment analysis. **A** Heatmap showing correlation coefficients between module eigengenes (MEs) and clinical characteristics including body composition (BMI, WHR, fat mass %), insulin-sensitivity (glucose levels and HOMA-IR), kidney function (mGFR; iohexol clearance), and blood pressure (systolic and diastolic). Positive correlations are represented in red, and negative correlations are represented in blue, with significance indicated by stars (*p < 0.05; **p < 0.01; ***p < 0.001). **A** Bar plot of gene counts within each ME. **B** Bar plots showing the enrichment of KEGG and Reactome pathways in the most significantly correlated MEs for fat mass % (ME3, ME4, ME9, ME14 and ME15) and HOMA-IR (ME4, ME7, ME14 and ME15). PRAT: *n* = 28

To further investigate how clinical characteristics are related to PRAT gene expression, we performed a WGCNA that identified multiple co-expressed gene modules (module eigengenes, MEs) (Fig. 6). Correlating these MEs with clinical traits revealed several significant positive and negative associations, particularly with body composition (fat mass %) and insulin sensitivity (HOMA-IR) (Fig. 6A). No significant associations were observed between the modules and body size (BMI), kidney function (mGFR), or blood pressure (systolic or diastolic). To provide a biological context, we performed functional enrichment analyses of the group of genes associated with body composition (fat mass %) and insulin sensitivity (HOMA-IR) (Fig. 6B and additional File 2 Table S10). Fat mass % was positively correlated with biological processes related to rRNA metabolic processes, ribosome biogenesis and immune responses. Conversely, fat mass % was negatively correlated with pathways involved in the degradation of valine, leucine and isoleucine and biological processes related to catabolic processes, metabolism, and cellular respiration. HOMA-IR was correlated with pathways similar to those correlated to body composition (fat mass %) and was also positively associated with KEGG pathways involved in the complement and coagulation cascades.

### Immune cell profiles across adipose tissue depots

**Fig. 7:**
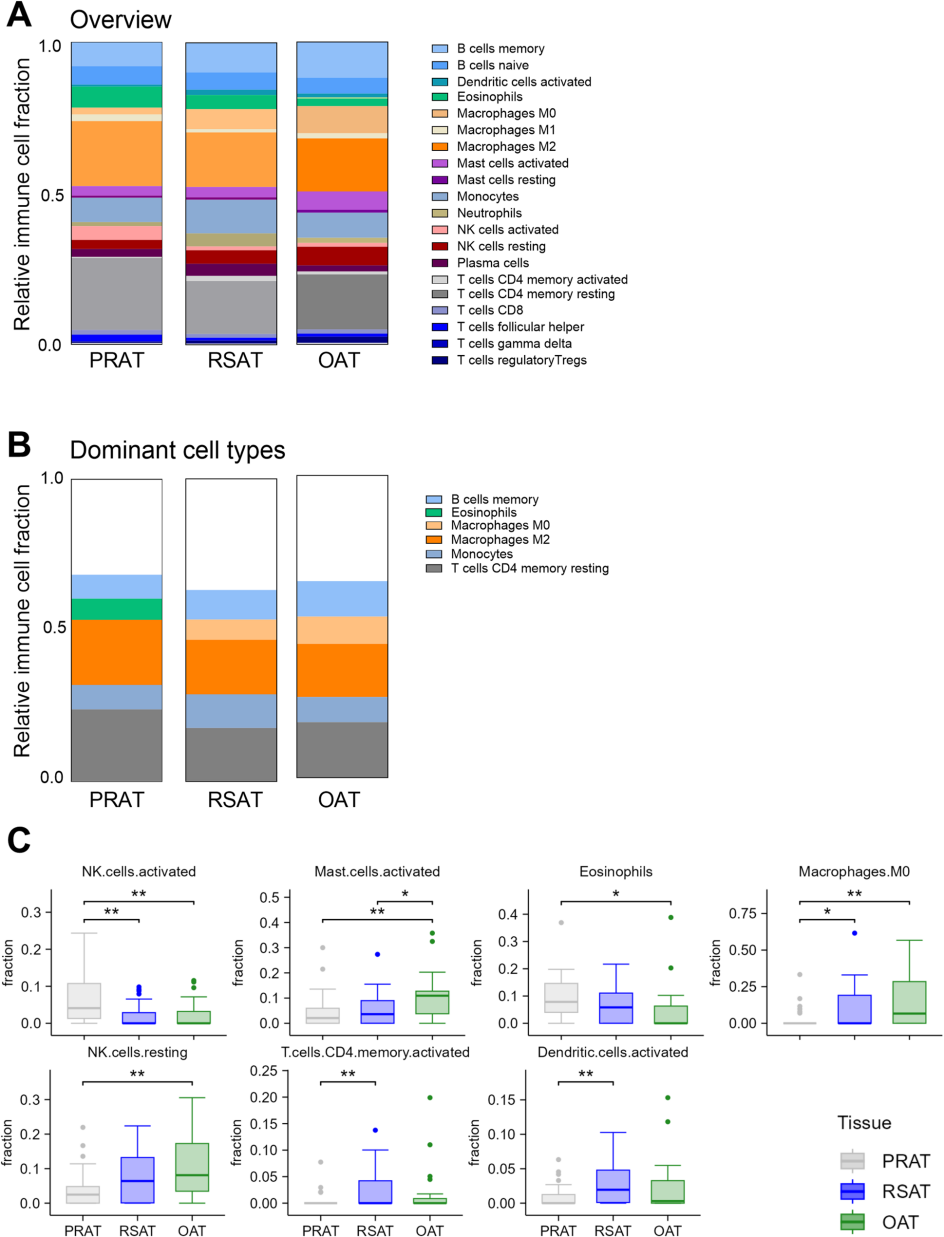
Estimated fractions of immune cells inferred from RNAseq deconvolution in perirenal adipose tissue (PRAT), renal sinus adipose tissue (RSAT), and omental adipose tissue (OAT). **A** Overview of the relative immune cell-type abundances across PRAT, RSAT, and OAT. Stacked bar plots display the normalized relative contributions of various immune cell gene signatures within each sample, such that the total immune cell fraction sums to 1. **B** The most prominent immune cell fraction across the three depots. **C** Significant differences in immune cell-type fractions estimates for activated NK cells, activated mast cells, eosinophils, M0 macrophages, resting NK cells, activated memory CD4 T cells and activated dendritic cells between the tissues (median ± IQR). PRAT: *n* = 28, RSAT: *n* = 28, OAT: *n* = 26. *p < 0.05; **p < 0.01

Immune cell deconvolution of the RNAseq data revealed distinct, depot-specific immune profiles across PRAT, RSAT, and OAT, with notable contributions from B cells, macrophages, monocytes, and CD4 T cells (Fig. 7A and additional File 2 Table S11). M0 and M2 macrophages, monocytes, resting memory CD4 T cells, and memory B cells were the most abundant fractions across depots (Fig. 7B). Depot-specific differences included a larger fraction of activated NK cells in PRAT compared to RSAT and OAT (*p* < 0.05) and eosinophils in PRAT compared to OAT (*p* < 0.05). OAT exhibited a greater fraction of activated mast cells and macrophages M0 compared to PRAT and RSAT (*p* < 0.05), and RSAT had a enhanced pro-inflammatory profile, including activated memory CD4 T cells and dendritic cells, than PRAT (*p* < 0.01) (Fig. 7C; Additional File 2 Table S12).

### Macrophage infiltration and pro-inflammatory expression across adipose tissue depots

**Fig. 8:**
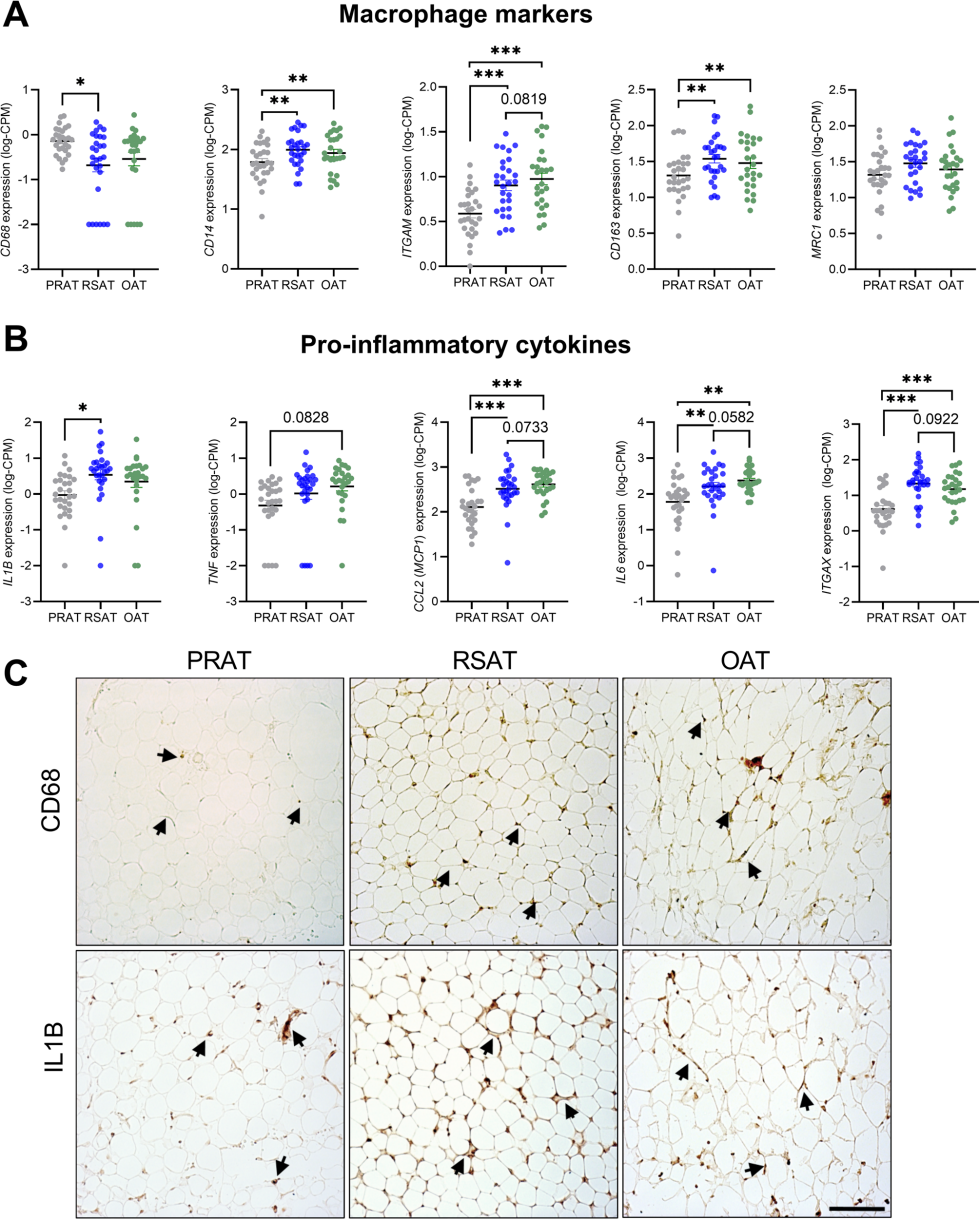
Macrophage and pro-inflammatory marker expression in PRAT, RSAT, and OAT (**A**) mRNA expression levels of the macrophage markers *CD68*,* CD14*,* ITGAM*,* CD163*, and *MRC1*. **B** mRNA expression levels of the pro-inflammatory markers *IL1B*,* TNF*,* CCL2*,* IL6*, and *ITGAX*. **C** Representative immunohistochemical staining for CD68 and IL1B in PRAT, RSAT, and OAT. Positive cells (brown) are indicated by arrows. Images were acquired at 20× magnification (scale bar = 100 μm) and are representative of two independent samples per depot; *p < 0.05, **p < 0.01; ***p < 0.001. CD14, cluster of differentiation 14; CD68, cluster of differentiation 68; CD14, cluster of differentiation 14; ITGAM, integrin alpha M; CD163, cluster of differentiation 163; MRC1, mannose receptor C-type 1; IL1B, interleukin 1 beta; TNF, tumor necrosis factor; CCL2, C-C motif chemokine ligand 2 (also known as MCP-1); IL6, interleukin 6; ITGAX, integrin alpha X

To assess macrophage infiltration and inflammation, we measured the mRNA expression of canonical macrophage markers (*CD68*,* CD14*,* ITGAM*,* CD163*, and *MRC1*) (Fig. 8A) and key pro-inflammatory factors (*IL1B*,* TNF*,* CCL2*,* IL6*, and *ITGAX*) (Fig. 8B) in the adipose tissue depots. The gene expression of the macrophage markers *CD14*,* ITGAM* and *CD163* was significantly lower in PRAT than in RSAT and OAT (*p* < 0.01), whereas the expression of *CD68* was higher in PRAT than in RSAT. Among the cytokines and chemokines, *IL1B* was lower in PRAT than in RSAT (*p* < 0.05), whereas *CCL2* (MCP-1), *IL6*, and *ITGAX* expression were lower PRAT, than in both RSAT and OAT (*p* < 0.01). Overall, gene expression levels were similar between RSAT and OAT. Notably, MRC1, a marker of alternatively activated macrophages, was not significantly different between the adipose tissue depots. Immunohistochemistry staining for CD68 and IL1B (Fig. 8C) supported the mRNA findings, PRAT containing fewer CD68 and IL1B positively stained cells than RSAT, whereas OAT displayed an intermediate pattern. For macrophage and pro-inflammatory gene markers, no consistent differences were observed between lean and overweight subjects (Additional File 1 Figure S8). However, there was a trend toward higher CD68 expression in OAT and TNF expression in PRAT among overweight individuals.

## Discussion

In this unique study, we comprehensively compared molecular, metabolic, and immune characteristics across three distinct adipose tissue depots; two kidney-related depots, perirenal adipose tissue (PRAT) and renal sinus adipose tissue (RSAT), and using omental adipose tissue (OAT) as a benchmark. PRAT exhibited a distinct thermogenic profile with elevated expression of BAT markers. Despite its anatomical proximity to RSAT, PRAT showed markedly different gene expression, with a more favourable metabolic and immune profile. This included reduced expression of inflammation-related genes, such as *NFKBIA*, *BIRC3*, and *IRF1* and enhanced activation of metabolic pathways, including the TCA cycle and oxidative phosphorylation, with upregulation of key metabolic genes such as *UCP1*, *CKMT1B*, and *FOXD1.* Comparisons between overweight and normal-weight individuals revealed that excess weight modifies PRAT and RSAT gene expression profiles by increasing the expression of inflammatory pathways and reducing metabolic pathways, underscoring the potential importance of weight-dependent mechanisms in kidney-related adipose tissue depots. The significant differences in adipocyte size across adipose tissue depots suggest functional specialization. The smaller adipocytes in PRAT, compared to those in OAT and SAT, likely reflect variations in lipid metabolism and storage capacity between the adipose tissue depots, aligning with previous reports of smaller adipocytes in kidney-related depots [5]. Positive associations between PRAT adipocyte size and insulin sensitivity, independent of RSAT, suggest that hypertrophy in PRAT is linked to metabolic dysfunction and cardiovascular risk [28]. The reduced significance after adjusting for OAT indicates that OAT may share overlapping metabolic pathways with PRAT or exert a stronger influence on insulin regulation. This depot-specific analysis highlights the importance of fat distribution in studies of metabolic function. While adipocyte diameter in PRAT, OAT, and SAT correlated positively with insulin levels and HOMA-IR, RSAT adipocyte size did not. This suggests that, unlike in other depots where adipocyte hypertrophy is linked to metabolic dysfunction, that in RSAT metabolic dysfunction may be driven more by inflammation. This interpretation aligns with our transcriptomic and immune cell analyses, which reveal enrichment of pro-inflammatory pathways and immune cell populations in RSAT.

The fold increase in adipocyte glucose uptake in response to insulin was approximately 1.5- to 3-fold, with PRAT exhibiting the highest insulin-stimulated uptake rate. This range is consistent with our previous findings and those of others, particularly for SAT and OAT [14, 29]. In line with this, PRAT also showed higher GLUT4 expression compared to RSAT and OAT, indicating a greater capacity for glucose disposal.

Our study reveals that PRAT has a distinct metabolic profile compared to RSAT and OAT, characterized by elevated expression of genes related to thermogenesis and energy metabolism, including UCP1 and other brown/beige adipocyte markers, e.g., *CIDEA* and *PRDM16*, aligning with previous studies [10, 30, 31]. The consistently higher *UCP1* expression indicates a robust brown/beige adipocyte-like phenotype in PRAT and highlights its unique role in thermogenesis. Additionally, PRAT showed increased expression of lipid metabolism-related genes, such as *FABP4* and *LPL*, suggesting a critical role in lipid handling and metabolic adaptation. Despite these thermogenic and metabolic features, PRAT displayed normal expression levels of white adipose tissue (WAT) markers, emphasizing the heterogeneity of this depot. Although RSAT is not classically thermogenic, sporadic peaks of UCP1 expression in tissue samples from RSAT, suggests inducible beige adipocyte activity. While prior rodent studies identified brown adipocytes in RSAT [30], further human validation is needed.

The higher expression of neurotrophic and neuronal markers (*CALCA*,* NGF*,* NTRK2*, and *UCHL1*) in OAT than in both PRAT and RSAT suggests greater innervation or neuronal activity. This aligns with OATs important role in metabolic regulation, particularly in lipolysis and stress response [32, 33]. Although direct comparisons of innervation density between human fat depots remain limited [34], our findings support the notion that OAT is more metabolically active and responsive to adrenergic stimulation [35]. Interestingly, despite PRAT being enriched with brown/beige adipocyte markers, it did not exhibit higher levels of neuronal markers than the other depots underscoring the need for further studies investigating innervation, neural density and activity across different adipose tissue depots and potential implications for metabolic regulation.

The differential gene expression analysis between PRAT and RSAT revealed distinct depot-specific metabolic and inflammatory features despite their anatomical proximity. PRAT is enriched in genes associated with mitochondrial function, energy metabolism, and thermogenesis, such as *UCP1*, *FOXD1*, *CKMT1B*, and *CYP1A2* [36–38], but has lower expression of inflammatory and immune-related genes, including *NFKBIA*, *BIRC3*, and *IRF1* [39–41]. This suggests that PRAT may have a protective metabolic role. RSAT, despite being extraperitoneal, resembled OAT in inflammation-related gene expression and was enriched in IL-17, TNF-α, and NF-κB signaling pathways [5]. Correlations between PRAT gene expression (e.g., *CKMT1A*,* CYP1A2*) and markers of insulin sensitivity and body composition further supports PRATs role in systemic metabolic regulation. Conversely, the association between RSAT and inflammatory markers along with correlations with insulin resistance and fat mass % (e.g., *BIRC3*,* BCL2A1*,* IRF1*, and *NFKBIA)* highlights a potential link to metabolic, cardiovascular and chronic kidney diseases with local and/or systemic inflammation as a common denominator and potential mediator.

Several studies have shown that obesity increases the volume of both PRAT and RSAT. PRAT expansion, in particular, has been linked to adverse renal outcomes [7]. In contrast, data on RSAT are limited. However, existing imaging suggests that RSAT volume is increased in individuals with obesity and hypertension and is associated with reduced kidney function [7, 42]. Due to its anatomical location, RSAT may affect the kidneys through both mechanical pressure and local inflammation. However, its inflammatory gene profile has not been previously studied in humans. Our study addresses this gap by providing the first transcriptomic and immune cell gene signature characterization of RSAT and PRAT in healthy individuals, laying the foundation for future research on their roles in obesity-related kidney disease.

Exploratory analyses revealed correlations between clinical parameters and depot-specific gene networks, even in this mostly normal-weight individuals. Significant associations were observed between body composition (fat mass %) and insulin sensitivity (HOMA-IR) and distinct gene networks. Fat mass % showed more robust associations with depot-specific gene expression than did BMI or WHR, suggesting it better reflects adiposity-driven molecular changes. These networks are linked primarily to extracellular matrix organization and immune responses, indicating a link between adipose tissue expansion, insulin resistance, and structural and inflammatory changes in PRAT [43]. Fat mass % was positively correlated with pathways related to ribosomal RNA expression and ribosome biogenesis, indicating an increased protein synthesis capacity within PRAT in response to metabolic challenges [44]. Conversely, fat mass % was negatively correlated with pathways involved in branched-chain amino acid (BCAA) catabolism, indicating altered metabolism that can lead to accumulation of BCAAs, linked to insulin resistance and metabolic inflexibility [45–47]. Additionally, fat mass % was negatively associated with pathways related to cellular respiration and the TCA cycle, suggesting a reduction in mitochondrial function as fat mass increases consistent with findings in other fat depots [48].

To evaluate the impact of excess weight on PRAT and RSAT, we conducted exploratory transcriptomic comparisons between overweight and normal-weight individuals. In PRAT, genes involved in actin cytoskeleton regulation and VEGF signaling were upregulated in overweight subjects, whereas pathways related to carbon metabolism, the TCA cycle, and oxidative phosphorylation were downregulated. In RSAT, Ras signaling and ribosome function were upregulated, while fatty acid metabolism and branched-chain amino acid degradation pathways were downregulated. These findings did not remain significant after FDR correction, emphasizing the exploratory nature of this analyses and the need for larger cohorts. Nonetheless, they align with previous findings from SAT and OAT regarding obesity-related traits [14, 49, 50]. Notably, the upregulation of Ras-related genes in RSAT is of interest due to Ras activation role in driving fibrosis, a key process in chronic kidney disease development [51]. Future studies in larger cohorts are warranted to investigate the role of Ras signaling in RSAT in obesity-related development of kidney disease.

Using deconvolution techniques we analyzed immune-related gene signatures from adipose tissue transcriptomic data [52]. PRAT showed unique immune characteristics, with enrichment in anti-inflammatory and homeostatic transcripts, including pathways for activated natural killer (NK)-like activity and eosinophil-related traits [53, 54]. Activated NK cells support immune surveillance and tissue homeostasis, whereas eosinophils promote type 2 immune responses that enhance insulin sensitivity and reduce inflammation [55]. PRAT also exhibited lower expression of inflammatory markers, i.e., macrophage-like activity [56], mast cell-like activation, resting NK cells [57] and antigen-presenting activity [58], indicating reduced inflammation. This immune-associated transcriptomic profile aligns with the broader molecular signature of PRAT, characterized by downregulated inflammation-related pathways but increased metabolic activity. Our findings are supported by direct assessments of macrophages and pro-inflammatory markers. mRNA expression of canonical macrophage markers (e.g., *CD14* and *ITGAM*) and inflammatory mediators (*IL1B*, *CCL2*, *IL6*, and *ITGAX*) was significantly increased in RSAT and OAT compared to PRAT, indicating greater immune filtration. Although CD163 is a commonly used M2 macrophage marker, its expression is not exclusive to this phenotype and can be influenced by the tissue microenvironment [59]; thus, its elevation in RSAT and OAT should be interpreted cautiously and validated with additional markers or assays. Immunohistochemistry for CD68 and IL1B corroborated these mRNA findings, such that RSAT had more cells positive for these markers, whereas PRAT had fewer. Taken together, these new results reinforce that PRAT has a more anti-inflammatory profile compared to RSAT and OAT, potentially contributing to its more favorable metabolic characteristics.

The strengths of this study include a well-phenotyped cohort and paired adipose tissue samples from up to four adipose tissue depots, a rare opportunity, especially in healthy individuals, made possible by the laparoscopic surgical approach used for kidney donation. However, some limitations should be acknowledged. The small sample size limits statistical power, and the cross-sectional design prevents establishing causation. The narrow range in body composition (fat mass percentage) limits evaluation of obesity’s impact on adipose tissue function. The inclusion of only healthy individuals limits the generalizability of the findings to other populations. While the study focused on depot-specific transcriptomic and immune differences, further mechanistic studies are needed to clarify immune-metabolic interactions in PRAT under varying physiological or pathological conditions. Immune characterization was based on bulk transcriptomic data, and future single-cell analysis are warranted. Additionally, subtle differences in vascularization or residual blood contamination cannot be excluded and may affect interpretation of immune cell profiles.

## Conclusions

In conclusion, this study highlights the functional heterogeneity between kidney-related perirenal (PRAT) and renal sinus adipose tissue (RSAT) depots, demonstrating unique depot-specific metabolic and immune/inflammatory profiles despite their anatomical proximity. PRAT is characterized by higher expression of thermogenic and mitochondrial markers and lower expression of immune-related genes compared with RSAT. Conversely, RSAT exhibits higher expression of macrophage markers, reflecting a more inflammatory phenotype. These findings underscore the importance of depot-specific adipose tissue characteristics, which may contribute to the development of obesity-related metabolic and cardiovascular and renal diseases.

## Supplementary Information

Supplementary Material 1.

Supplementary Material 2.

## Data Availability

The transcriptomics data supporting the findings of this study are openly available in Array Express at E-MTAB-13869. Additional clinical and adipocyte data are not publicly available but can be provided from the corresponding author upon reasonable request.

## Abbreviations

BAT: Brown adipose tissue
CKD: Chronic kidney disease
DEGs: Differentially expressed genes
eGFR: Estimated glomerular filtration rate using creatinine
GO: Gene ontology
GSEA: Gene set enrichment analysis
ME: Module eigengenes
mGFR: Measured glomerular filtration rate using iohexol clearance
OAT: Omental adipose tissue
PRAT: Perirenal adipose tissue
RPVAT: Renal perivascular adipose tissue
RSAT: Renal sinus adipose tissue
SAT: Subcutaneous adipose tissue
TCA: Citric acid cycle
WAT: White adipose tissue
WGCNA: Weighted gene co-expression network analysis
