# Supplementary material for: Depot-specific metabolic and inflammatory profiles in perirenal and renal sinus adipose tissue
Source: Mol Med. 2025 Jul 22;31:262. doi: 10.1186/s10020-025-01323-1 (PMC12285007; doi:10.1186/s10020-025-01323-1)
Supplement: Supplementary file 1 — Supplementary Material 1. [file 10020_2025_1323_MOESM1_ESM.docx]

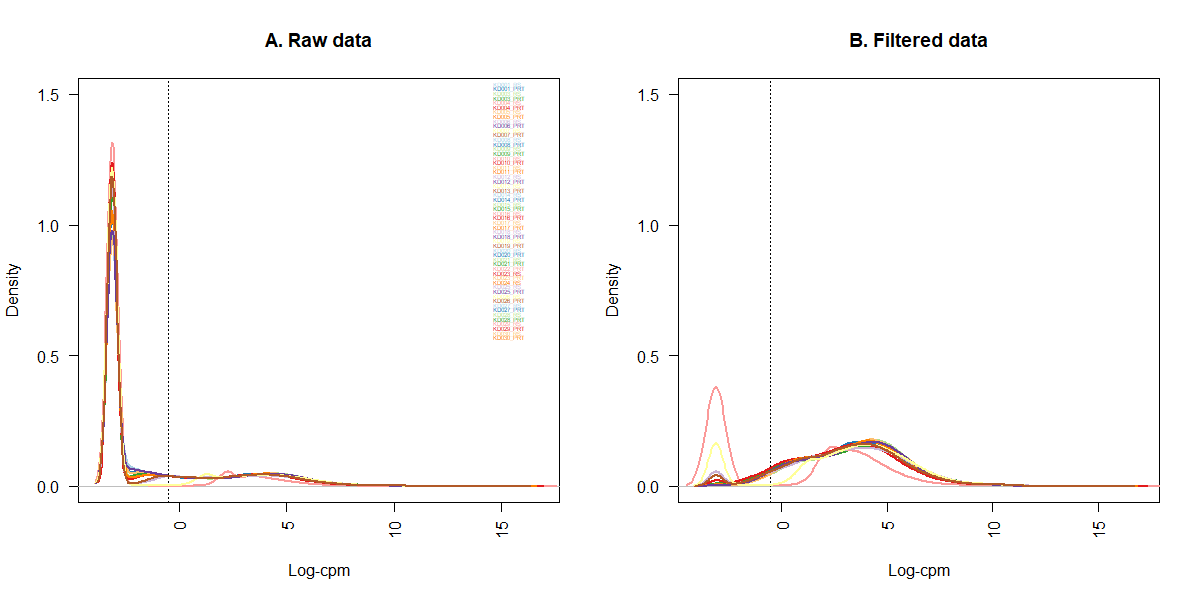


**Figure S1.** Log-CPM Distribution Before and After Filtering. The density of log-CPM values for raw pre-filtered data (A) and post-filtered data (B) are shown for each sample. Dotted vertical lines mark the log-CPM threshold used in the filtering step.


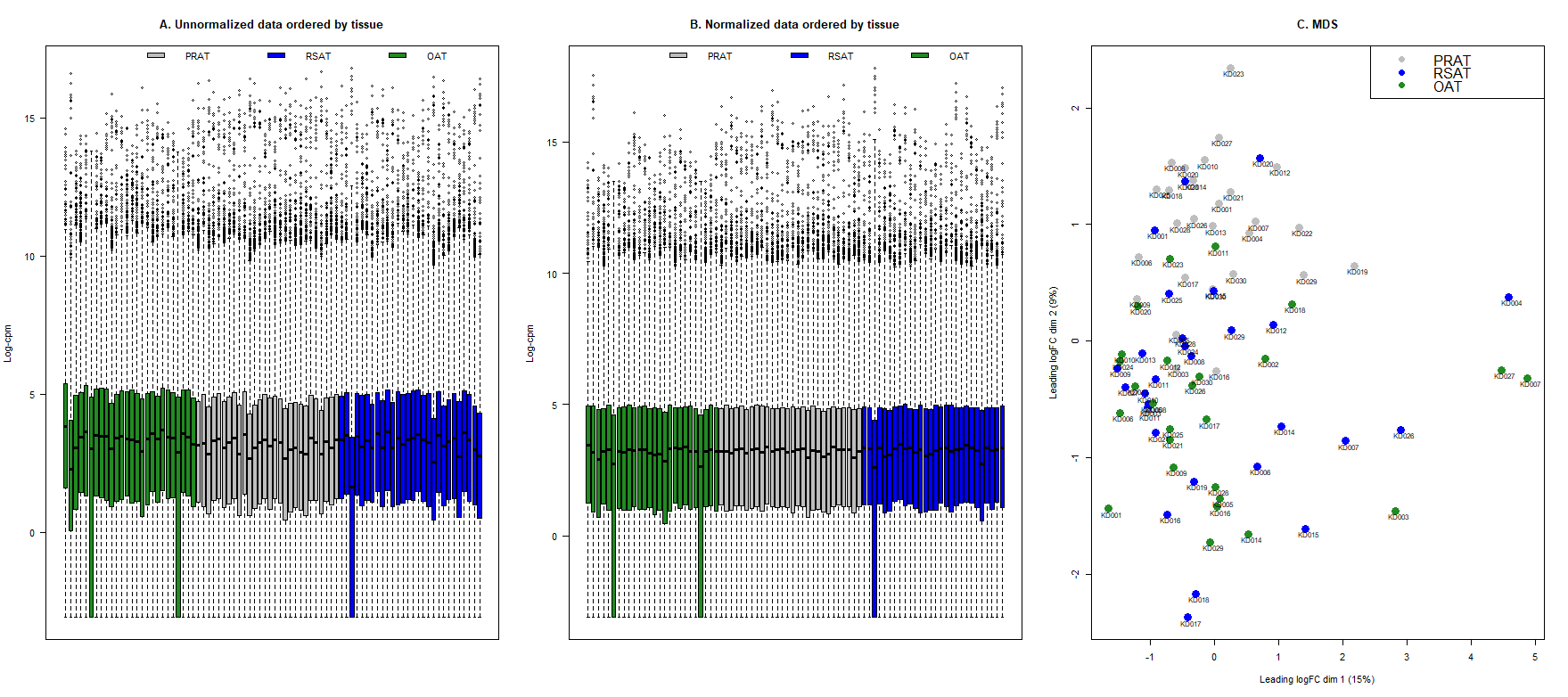


**Figure S2.** Expression distributions for transcriptomic data and MDS. Boxplots of log-CPM values in unnormalized (A) and normalized data (B) of the kidney donor dataset in omental (OAT: green), renal sinus (RSAT: blue), and perirenal (PRAT: grey) adipose tissues. MDS plots of log-CPM values over dimensions 1 and 2 with samples colored by the tissue type. The first dimension represents the leading-fold-change that best separates samples and explains the largest proportion of variation in the data. The distances on the plot correspond to the leading fold-change, which is the average (root-mean-square) log2-fold-change for the 500 genes most divergent between each pair of samples by default.


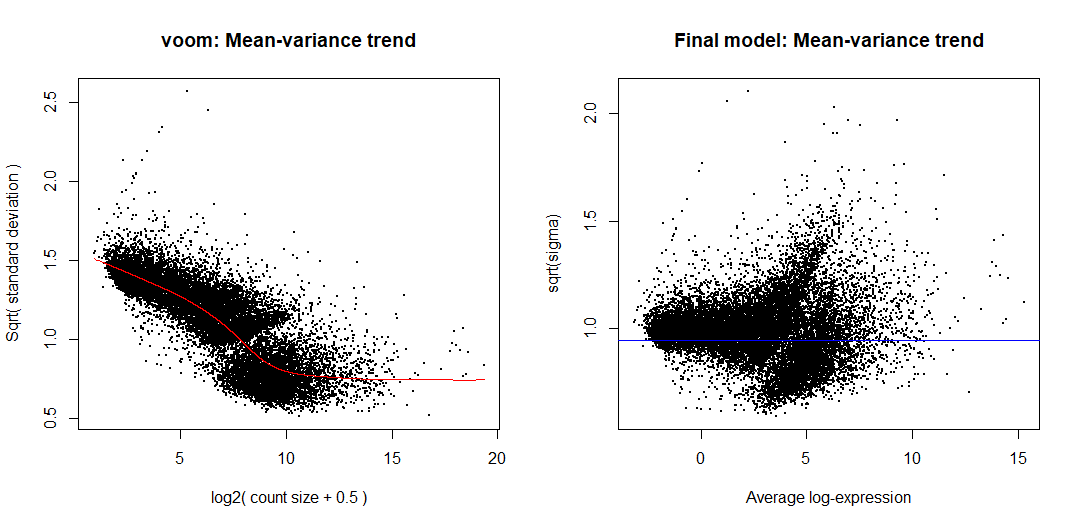


**Figure S3.** Mean variance trend before and after voom correction. Means (x-axis) and variances (y-axis) of each gene are plotted to show the dependence between the before voom is applied to the data (left panel) and how the trend is removed after voom precision weights are applied to the data (right panel).


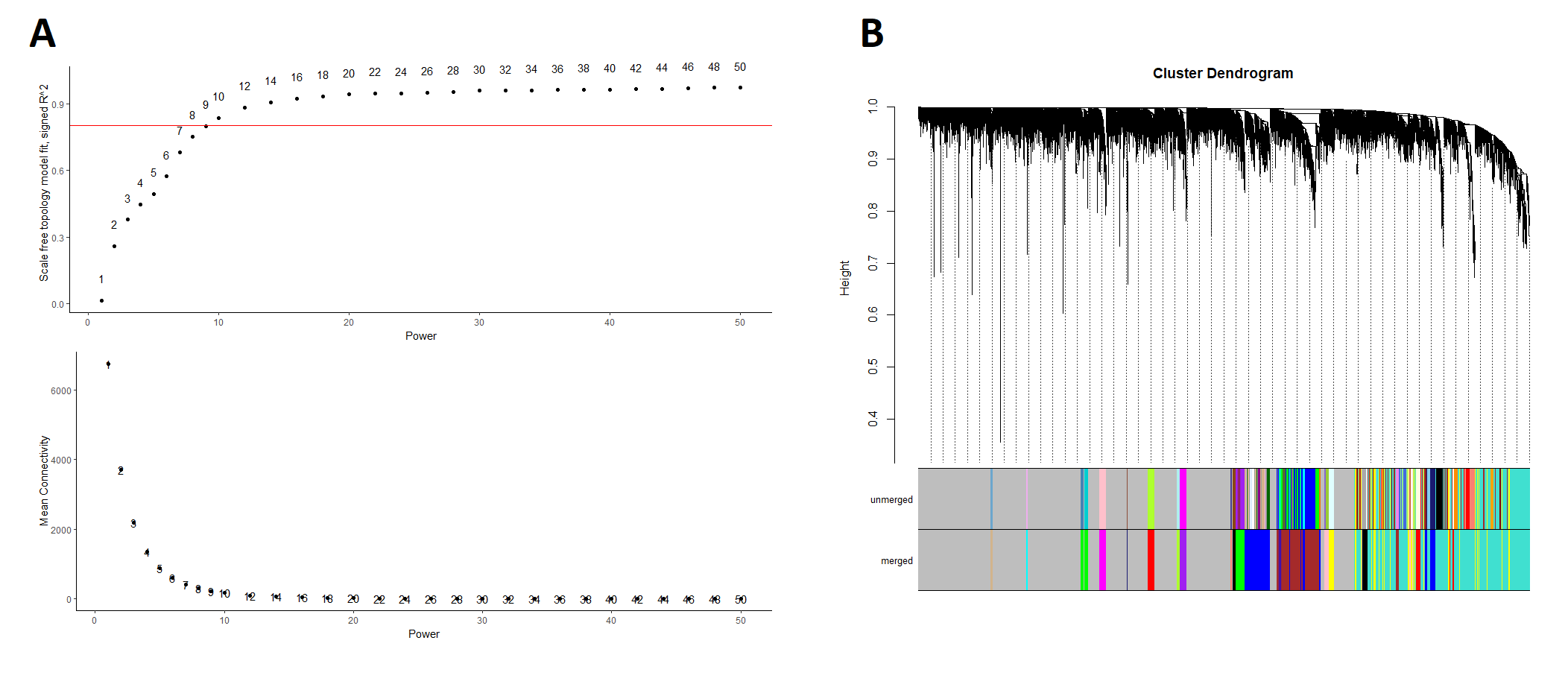


**Figure S4.** Soft threshold plots (scale-free topology and mean connectivity) and dendrogram for the module eigengenes revealed from the WGCNA.


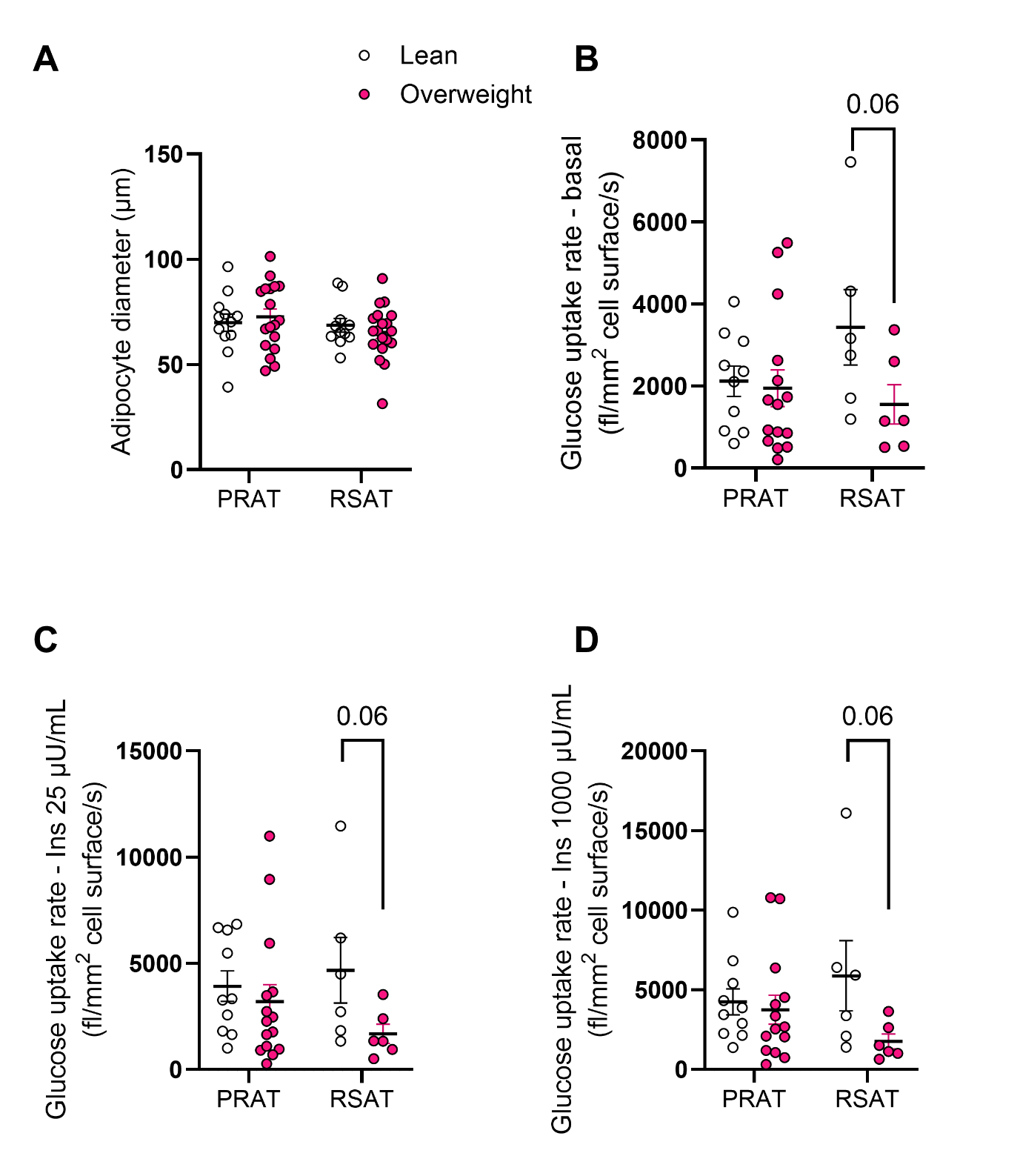
**Figure S5. Adipocyte size and glucose uptake rates in perirenal (PRAT) and renal sinus (RSAT) adipose tissue from lean and overweight individuals. (A) Adipocyte diameter (µm) measured in PRAT and RSAT. (B) Basal glucose uptake rate. (C) Glucose uptake rate in response to 25 µU/mL insulin. (D) Glucose uptake rate in response to 1000 µU/mL insulin. Open circles represent lean individuals (PRAT: n= 10; RSAT: n=6); magenta circles represent overweight individuals (PRAT: n=15; RSAT: n= 6). p-values represent trends toward significance (p = 0.06).**


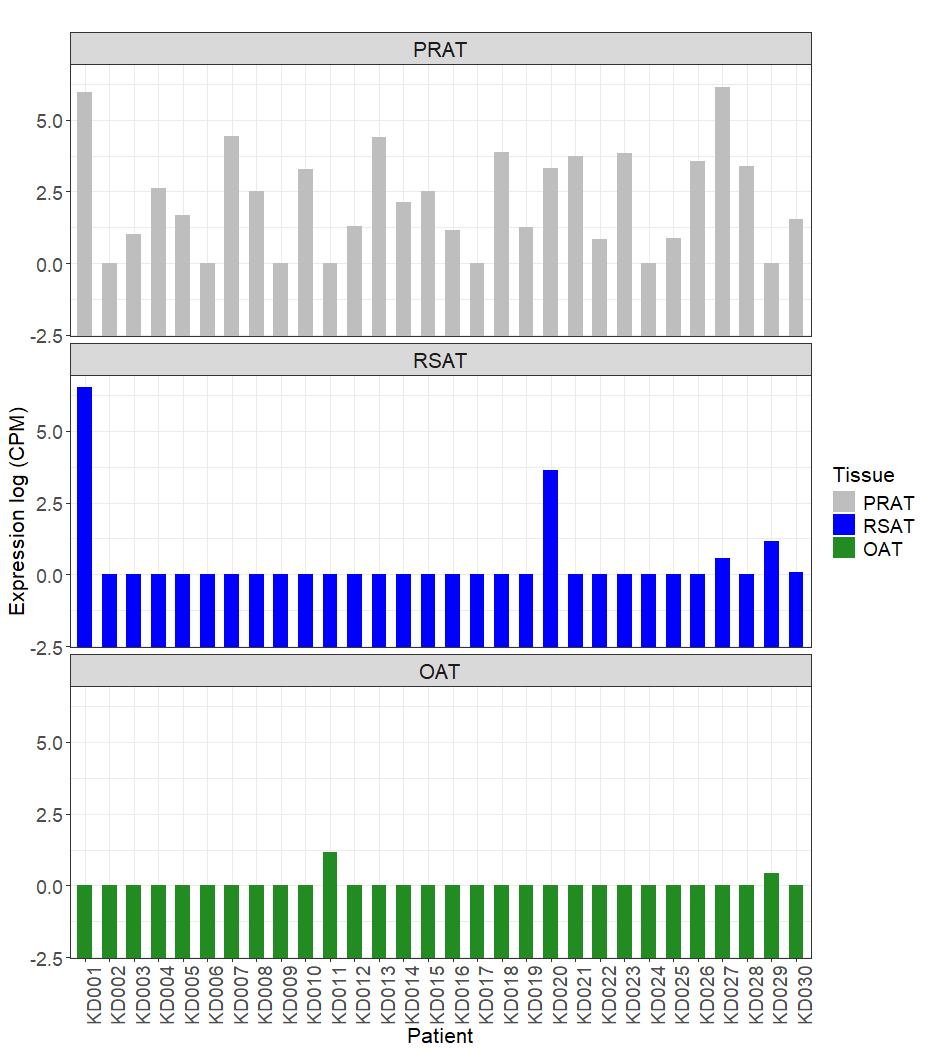

**Figure S6.** UCP1 expression across paired individual samples by adipose tissue depot; perirenal adipose tissue (PRAT), renal sinus adipose tissue (RSAT), and omental adipose tissue (OAT). (A) The expression levels of UCP1, a key marker of thermogenic brown adipose tissue, are shown as log counts per million (logCPM) for individual samples across three adipose tissues: perirenal adipose tissue (PRAT, gray), renal sinus adipose tissue (RSAT, blue) and omental adipose tissue (OAT, green). Each dot represents a single sample, and the connecting lines indicate the variation in UCP1 expression within each depot. B) immunohistochemistry for UCP1 in the three depots.


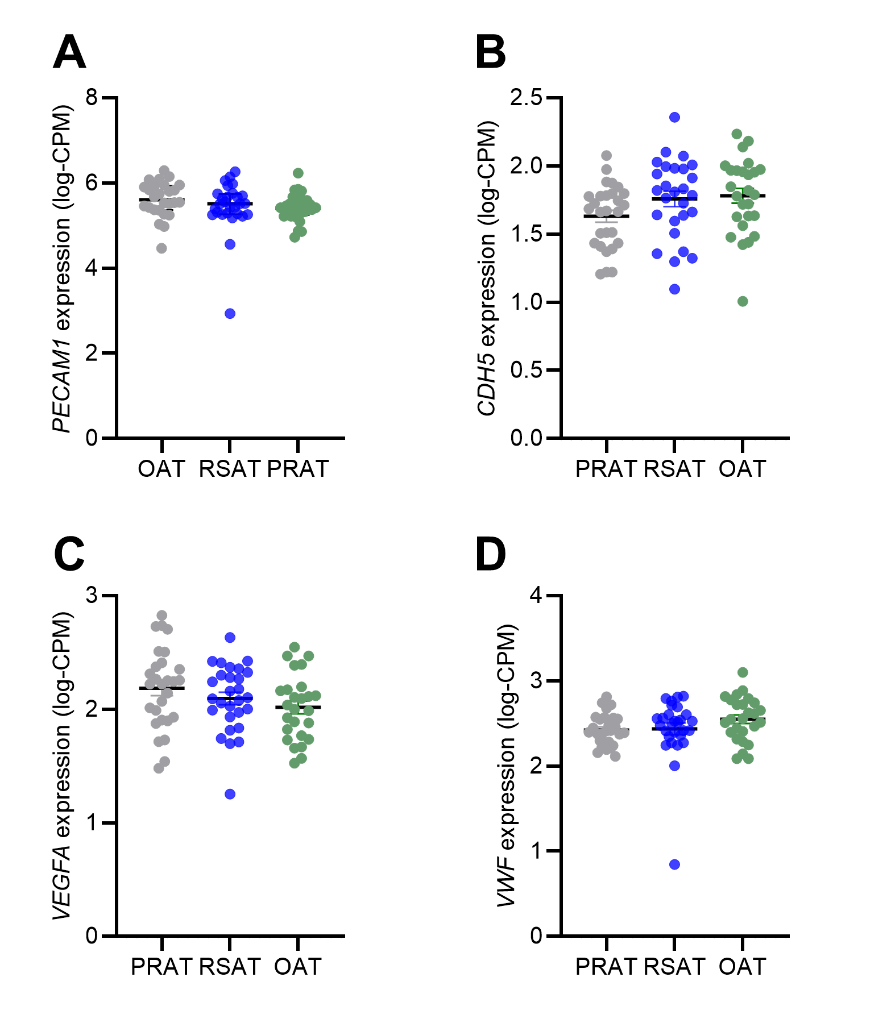


**Figure S7.** **Expression of vascular markers across adipose tissue depots.**Expression levels of key vascularization markers (A) ***PECAM1*** *(*platelet/endothelial cell adhesion molecule 1*, CD31),* (B) ***CDH5*** *(*cadherin 5*, VE-cadherin, CD144),* (C) ***VEGFA*** *(*vascular endothelial growth factor A*), and* (D) ***VWF*** *(*von Willebrand factor*)* in perirenal (PRAT), renal sinus (RSAT), omental (OAT) adipose tissue samples. No significant differences in expression were detected between the depots. PRAT: n=28, RSAT: n=28, OAT: n=26.


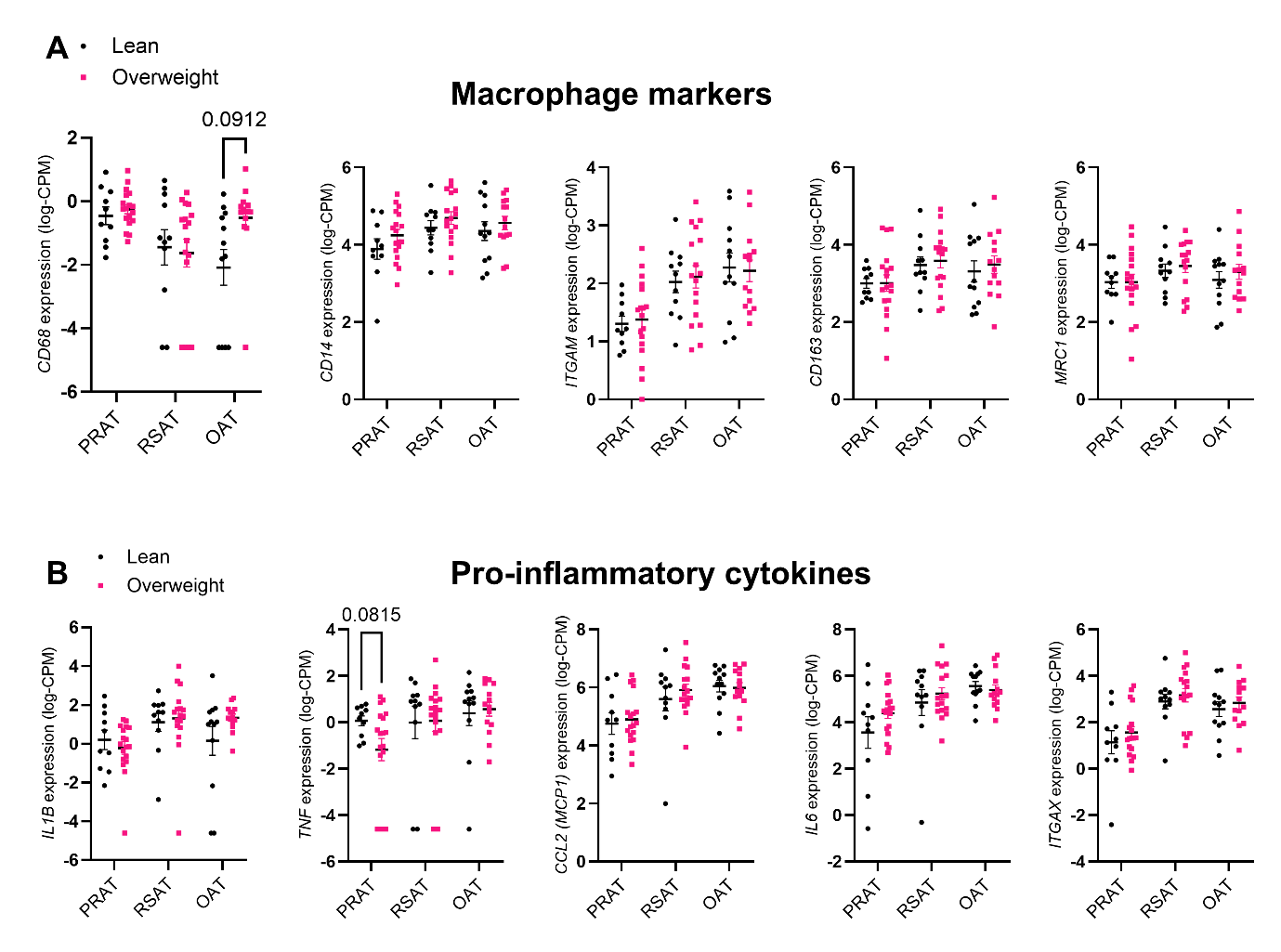


**Figure S8.** Expression of macrophage and pro-inflammatory markers in adipose tissue depots from lean and overweight individuals. (A) mRNA expression levels of macrophage-associated genes CD68, CD14, ITGAM, CD163, and MRC1 in perirenal (PRAT), renal sinus (RSAT), and omental (OAT) adipose tissue. (B) mRNA expression levels of pro-inflammatory markers IL1B, TNF, CCL2, IL6, and ITGAX in the same depots. Lean group: PRAT (n = 10), RSAT (n = 11), OAT (n = 12); Overweight group: PRAT (n = 18), RSAT (n = 17), OAT (n = 14).
